# New Knowledge on Distribution and Abundance of Toxic Microalgal Species and Related Toxins in the Northwestern Black Sea

**DOI:** 10.3390/toxins14100685

**Published:** 2022-10-06

**Authors:** Nina Dzhembekova, Snejana Moncheva, Nataliya Slabakova, Ivelina Zlateva, Satoshi Nagai, Stephan Wietkamp, Marvin Wellkamp, Urban Tillmann, Bernd Krock

**Affiliations:** 1 Institute of Oceanology “Fridtjof Nansen”—Bulgarian Academy of Sciences, 9000 Varna, Bulgaria; 2 Fisheries Research and Education Agency, Fisheries Technology Institute, Yokohama 236-8648, Kanagawa, Japan; 3 Alfred Wegener Institut-Helmholtz Zentrum für Polar- und Meeresforschung, Ökologische Chemie, 0471 Bremerhaven, Germany

**Keywords:** toxic microalgae, light microscopy, metabarcoding, phycotoxins, Black Sea

## Abstract

Numerous potentially toxic plankton species commonly occur in the Black Sea, and phycotoxins have been reported. However, the taxonomy, phycotoxin profiles, and distribution of harmful microalgae in the basin are still understudied. An integrated microscopic (light microscopy) and molecular (18S rRNA gene metabarcoding and qPCR) approach complemented with toxin analysis was applied at 41 stations in the northwestern part of the Black Sea for better taxonomic coverage and toxin profiling in natural populations. The combined dataset included 20 potentially toxic species, some of which (*Dinophysis acuminata*, *Dinophysis acuta*, *Gonyaulax spinifera*, and *Karlodinium veneficum*) were detected in over 95% of the stations. In parallel, pectenotoxins (PTX-2 as a major toxin) were registered in all samples, and yessotoxins were present at most of the sampling points. PTX-1 and PTX-13, as well as some YTX variants, were recorded for the first time in the basin. A positive correlation was found between the cell abundance of *Dinophysis acuta* and pectenotoxins, and between *Lingulodinium polyedra* and *Protoceratium reticulatum* and yessotoxins. Toxic microalgae and toxin variant abundance and spatial distribution was associated with environmental parameters. Despite the low levels of the identified phycotoxins and their low oral toxicity, chronic toxic exposure could represent an ecosystem and human health hazard.

## 1. Introduction

During the past few decades, problems related to harmful algal bloom (HAB) events have been observed globally [1,2,3]. Negative effects occur as a consequence of the potent toxins produced by certain microalgal species that affect human and ecosystem health [4,5]. The range of phycotoxins is rather extensive, and the number of reported toxins and toxic species continues to increase [6]. On a worldwide basis, marine algal toxins are responsible for more than 60,000 human intoxication incidents per year, with an overall mortality rate of 1.5% [7]. In addition, they cause mortalities among wild fish, seabirds, marine mammals, and other marine animals [4,8,9,10,11]. Chronic toxin exposure may have long-term consequences that are critical to human health and the sustainability or recovery of natural populations [4,5,12]. Toxic marine microalgae are associated with the production of numerous phycotoxins related to different types of poisoning syndromes—paralytic shellfish poisoning (PSP), diarrhetic shellfish poisoning (DSP), neurotoxic shellfish poisoning (NSP), amnesic shellfish poisoning (ASP), and azaspiracid shellfish poisoning (AZP) [13]. The types of poisoning and causative species are region- specific [3].

The Black Sea is a specific marine basin, largely isolated from the global ocean, and is characterized by extensive freshwater influx, strong vertical stratification, low salinity, and anoxic conditions below depths of 150–200 m [14]. It is surrounded by six countries, but influenced by 17 countries through the discharge of the major European rivers; Danube, Dnieper, and Don [15]. The Black Sea has been strongly affected by eutrophication in the past, and phytoplankton blooms along with an increase in dinoflagellate proportions have been of major concern to the health of the Black Sea ecosystem [16,17]. Although the conditions have been improved after 1992 [18], its ecological state is still unstable [17] and requires rigorous monitoring [19]. A total number of 49 species were listed in the “Atlas of toxic microalgae of the Black Sea and the Sea of Azov” [20], and the number has been continuously increasing [19], especially with the application of novel research techniques [21,22]. A large proportion of the potentially toxic species are common in the Black Sea plankton community, and some of them develop in bloom abundancies [17,19]. Even though the data on phycotoxins in the Black Sea are scarce and fragmentary [19], toxicity linked to microalgal species has been confirmed. Several investigations have been carried out along the Bulgarian and Russian coast. Domoic acid (DA) has been detected in mussel and plankton samples from Bulgarian waters [23,24,25], as well as in cultures of *Pseudo-nitzschia calliantha* isolated from Sevastopol Bay, Black Sea [26]. DSP toxins (okadaic acid—OA, dinophysistoxins—DTX-1 and DTX-2, and pectenotoxins—PTX-2 and PTX-2sa) have been found in farmed mussels and plankton samples in Russian waters in the presence of *Dinophysis caudata*, *Phalacroma rotundatum*, and *Prorocentrum lima*, and DSP cases have been observed in parallel [27,28]. In addition, yessotoxins have been detected in farmed mussels with the concomitant occurrence of *Lingulodinium polyedra* and *Gonyaulax spinifera* [29]. PSP toxins (saxitoxin—STX, B1, and gonyautoxins—GTX-2/3) were also reported in farmed mussels with the attendant presence of *Alexandrium* spp. in the samples [30]. Studies on the toxicity of plankton and shellfish samples from the Bulgarian Black Sea coast reported PSP toxins (STX, B1, and GTX-2/3) [25,31,32,33] and DSP toxins (YTX and PTX-2) [24,25], although without analytical investigations for the determination of the source of the toxins. Phycotoxicity (*Prymnesium parvum*-associated) has been proposed as a cause of fish mortality along the Bulgarian coast [34]. At the basin scale, there is still a lack of in-depth knowledge of the taxonomy, toxicity, and distribution of harmful microalgae in the Black Sea, calling for targeted investigations [19].

The aim of this study was to provide new data and insight into the presence (distribution and abundance) of potentially toxic plankton speciesand the associated phycotoxins in the western/northwestern part of the Black Sea. An integrated approach (combining morphological and molecular tools) complemented with toxin analysis was applied in order to assure better taxonomic coverage and toxin profiles in natural populations. The investigated area covered coastal, shelf, and open water stations, including waters affected by the Danube river flow, which has been identified as the main source of eutrophication in the western Black Sea [35]. Gathering data about harmful species abundance, distribution, and toxicity at a regional level is crucial for the development and implementation of effective monitoring programs and early warning systems [3,36].

## 2. Results

### 2.1. Environmental Characteristics of the Sampling Sites

**Figure 1:**
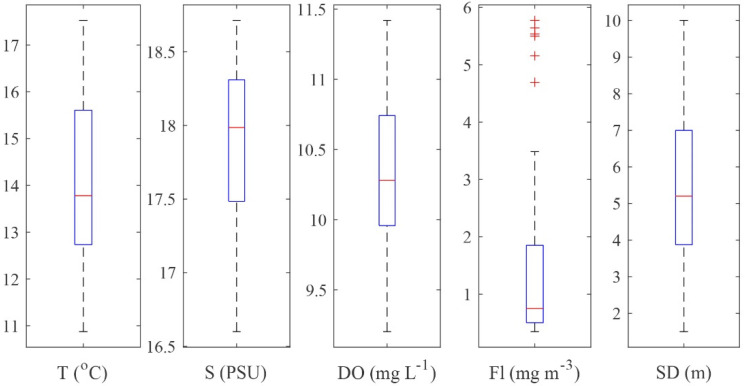
Boxplots of environmental variables (average value for the sampling layer): T, temperature (°C); S, salinity (PSU); DO, dissolved oxygen (mg L^−1^); Fl, fluorescence (mg m^−3^); SD, water transparency—Secchi depth (m).

The ranges of all oceanographic parameters of the survey are plotted in Figure 1, and detailed data are compiled in Supplementary Material Table S1. Surface seawater temperature increased during the survey period, starting on 15th May (12.2 °C; st. 1), with a peak registered on 3rd June (21.5 °C; st. 40). Sampling layer bottom temperature did not follow a similar pattern; it was more homogenous, with closer minimum and maximum values (from 8.3 °C at st. 38 to 12.3 °C at st. 32). On average, the stations sampled at the beginning of the campaign, as well as the stations with deeper sampling layers, had lower water column temperatures compared to the other stations. Surface seawater salinity ranged between 10.9 (st. 19) and 18.9 (st. 36), with lower values (<16) at the stations located in the northernmost transect (st. 16, 18–20). Sampling layer bottom salinity was relatively uniform, with a narrow range of 17.9 (st. 20) to 19.1 (st. 38). Coastal stations impacted by freshwater input were characterized by lower average salinity levels than open sea stations. Oxygen concentrations at the surface ranged from 8.83 mg L^−1^ (st. 27) up to a maximum of 12.17 mg L^−1^ (st. 12). Sampling layer bottom oxygen had similar minimum values (8.45 mg L^−1^; st. 33) and slightly lower maximum values (11.23 mg L^−1^; st. 32). Accordingly, the average values for the sampling layer were comparable (between 9.20 and 11.42 mg L^−1^). Fluorescence (average sampling layer levels) fluctuated between 0.35 (st. 27 and 29) and 5.77 mg m^−3^ (st. 20). Highest values (≥4.7 mg m^−3^) were found at stations with low average salinities ≤17.4 (stations 6–7 and 17–20) (the outliers in the boxplot in Figure 1). The water transparency (SD) ranged between 1.5 m (st. 18) and 10 m (st. 3), with the lowest Secchi depths (≤2.5 m) measured at coastal stations in Romanian waters (st. 7, 18–21).

### 2.2. Microscopy Identification and Enumeration of Potentially Toxic Microalgae

The microscopy analysis identified 12 putative toxic plankton taxa, out of which nine were identified at the species level (Supplementary Material Table S2). The diatom genus *Pseudo-nitzschia* dominated in terms of frequency (found at all stations) and abundance (accounting for more than 99.5% of the total abundance of the potentially toxic microalgae). Cells of the *P. delicatissima* group and *P. seriata* group co-occurred in most samples with maximum abundances of 375 × 10^6^ and 141 × 10^6^ cells NT^−1^ measured at station 40. Within the group of potentially toxic microalgae, there were more species of dinoflagellates than diatoms, but the former were much less abundant. Potentially toxic dinoflagellates were dominated by the genera *Prorocentrum*, *Dinophysis*, and *Alexandrium*, accounting for almost 87% of the total abundance of potentially toxic dinoflagellates. *Alexandrium* spp. were present at all analyzed stations in abundances ranging from 7 × 10^3^ (st. 35) to 242 × 10^3^ cells NT^−1^ (st. 18). High variability in cell size was observed, indicating that different species were present (Supplementary Material Table S2). Spatially, the genus was found in highest densities at the Romanian shelf stations (st. 18 and 22). *Dinophysis* was also detected across the entire sampling area, represented by four potentially toxic species. *Dinophysis acuminata* and *D. acuta* were more frequent, present in 98% of the samples. Cell abundances ranged from not observed (st. 36) to 990 × 10^3^ cells NT^−1^ (st. 20) for *D. acuminata*, and between not observed (st. 35) and 238 × 10^3^ cells NT^−1^ (st. 22) for *D. acuta*. *Dinophysis caudata* and *D. sacculus* were sporadic in their occurrence, with very low abundances. *Phalacroma rotundatum* was present in 83% of the samples, albeit only at low cell densities. *Gonyaulax spinifera*, *Lingulodinium polyedra*, and *Protoceratium reticulatum* were widespread within the study area (found in more than 70% of the samples), detected in variable abundances, ranging up to 110 × 10^3^ cells NT^−1^ for *G. spinifera* (st. 20), 132 × 10^3^ cells NT^−1^ for *L. polyedra* (st. 40), and 26 × 10^3^ cells NT^−1^ (st. 4 and 33) for *P. reticulatum*. Highest densities of *G. spinifera* and *L. polyedra* were detected at the coastal stations (st. 20 and 33 and st. 32 and 40, respectively), whereas *P. reticulatum* was most abundant both at coastal and open sea stations (st. 4 and 33). *Prorocentrum cordatum* was ubiquitous (present in 93% of the samples), with a peak of 2000 × 10^3^ cells NT^−1^ recorded at st. 18.

### 2.3. Potentially Toxic Species Detected with Metabarcoding

In total, the next generation sequencing (NGS) dataset from the 40 analyzed samples comprised 27 operational taxonomic units (OTUs) assigned to 18 potentially toxic plankton species (Supplementary Material Table S3). Dinoflagellates were the most diverse group in terms of toxic species. The highest OTU diversity was observed within the genus *Alexandrium* (seven OTUs in total). Three of the OTUs were clearly distinguished at the species level, associated with *A. andersonii* (identified at 11 stations), *A. minutum* (18 stations), and *A. ostenfeldii* (23 stations), all of which were detected with a small number of reads at the positive stations. Among the four remaining OTUs assigned to *Alexandrium*, three showed the same similarities with two different species (*A. pseudogonyaulax*/*A. hiranoi*; *A. tamarense*/*A. catenella*; *A. minutum*/*A. tamutum*) and one was assigned to a reference sequence deposited as *Alexandrium* sp.; all were pooled as *Alexandrium* spp. In addition, one non-toxic member, *A. margalefii*, was detected in all samples with significant sequence read numbers (not included in the dataset). Two of the dominant OTUs detected at all stations were affiliated with *Gonyaulax spinifera* and *Karlodinium veneficum*. The OTU annotated as *Protoceratium reticulatum* was widely distributed, being recorded at most of the sampling sites (83%) with fluctuating sequence numbers, whereas the OTU assigned to *Lingulodinium polyedra* was scarce, being recorded at only 33% of the stations with low sequence reads. *Dinophysis* was represented by four OTUs, two of which were clearly identified at the species level (associated with *D. acuta* and *D. acuminata*), and the other two shared similarities with multiple *Dinophysis* species (and were thus merged as *Dinophysis* spp.). The genus was widespread, with *D. acuta* and *D. acuminata* found in 80% and 58% of samples, respectively. Within the two OTUs assigned to *Prorocentrum*, only one was defined at the species level, allocated to *Prorocentrum cordatum* (detected at seven stations), while the other OTU, accepted at the genus level due to the same similarity with reference sequences of multiple potentially toxic *Prorocentrum* species, was more frequently found in the sampling area (35 stations). The other OTUs affiliated with potentially toxic dinoflagellates (*Amphidoma languida*, *Phalacroma rotundatum*, *Gymnodinium catenatum*, and *Polykrikos hartmannii*) were unevenly distributed across the stations, generally with a small number of sequences (Supplementary Material Table S3).

Diatoms were represented by five OTUs assigned to three potentially toxic *Pseudo-nitzschia* species. The majority of the sequences (three OTUs) were assigned to *P. calliantha*, present in 80% of the samples, whereas the OTUs associated with *P. delicatissima* and *P. pungens* appeared sporadically.

Among potentially toxic pelagophytes, only *Aureococcus anophagefferens* was detected at two stations, with a single sequence per sample.

### 2.4. qPCR Analyses of Field Samples

Of the 41 DNA samples from Niskin bottles tested with the amphidomatacean SYBR Green qPCR assay, samples from 11 stations (st. 2–5, 12–18; Supplementary Material Table S4) passed the evaluation process and revealed the presence of target DNA. However, no amplification was observed in any of these 11 samples in the three species-specific TaqMan assays targeting *Az. spinosum*, *Az. poporum*, and *Am. languida*. Thus, based on qPCR analysis, Amphidomataceae DNA in general was present in the samples, but none of the three specifically targeted AZA-producing species were detected.

### 2.5. Toxin Distribution

**Figure 2:**
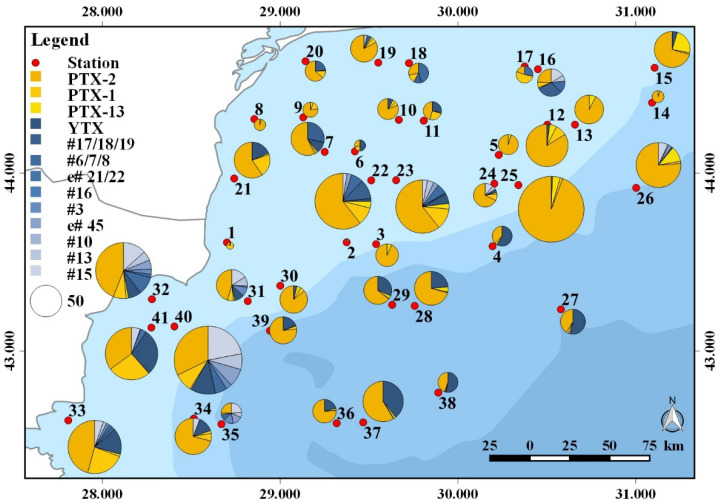
Geographic distribution and abundance (ng NT^−1^) of toxin variants in the 20–50 µm size fraction (toxin variant abbreviations are listed in Table S6).

**Figure 3:**
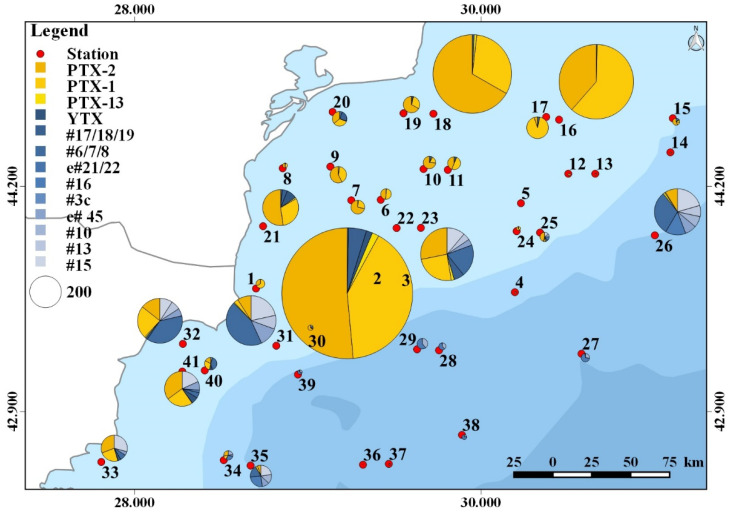
Geographic distribution and abundance (ng NT^−1^) of toxin variants in the 50–200 µm size fraction (toxin variant abbreviations are listed in Table S7).

Plankton samples were analyzed for a wide array of phycotoxins. With respect to paralytic shellfish toxins (PST), domoic acid (DA), azaspiracids (AZA), cyclic imines (gymnodimines, pinnatoxins, and spirolides), goniodomins, karlotoxins (KmTx), okadaic acid, and dinophysistoxins, none were detected in the planktonic field samples of the survey. The respective detection limits are provided in the supplementary information (Supplementary Material Table S5). In contrast to the absence of these groups of phycotoxins, pectenotoxins (PTXs) and yessotoxins (YTXs) were detected. Among PTXs (for structures see Supplementary Material Figure S1), the most frequently occurring variant in terms of total abundance and geographic distribution was PTX-2, of which the highest levels detected during this survey were 206 ng NT^−1^ in the 20–50 µm size fraction (Figure 2; Supplementary Material Table S6) of st. 25, and up to 426 ng NT^−1^ in the 50–200 µm size fraction (Figure 3; Supplementary Material Table S7) of st. 22. In addition to PTX-2, PTX-1 was also detected at high abundances, with maxima of 37 ng NT^−1^ in the 20–50 µm size fraction of st. 41 and 336 ng NT^−1^ in the 50–200 μm size fraction of st. 22. Both PTXs were generally found at higher abundance in the 50–200 µm fraction than in the 20–50 µm fraction. The third PTX detected in this survey was PTX-13, which, in general, was the least abundant PTX, with maxima of 15 and 14 ng NT^−1^ in the 20–50 and the 50–200 µm size fractions of st. 15 and 22, respectively (Figure 2 and Figure 3; Supplementary Material Tables S6 and S7). In contrast to PTX-1 and PTX-2, PTX-13 was more abundant in the 20–50 µm fraction than in the 50–200 µm fraction.

The other phycotoxin group present in the field sample of this study was the yessotoxin (YTX) group (for structures see Supplementary Material Figure S2), out of which 10 variants were detected in total. In addition to the base compound yessotoxin (YTX), nine other YTX variants were detected in the field samples. Among these variants were one of the three nor-ring A isomers 41-keto-, 40-epi-41-keto-, or 41-keto-enone-nor-ring A YTX (compounds #17/18/19 in [37]) with a molecular weight of 992 Da, as well as one of the three isomers 41-keto-, 40-epi-41-keto-, or 41-keto-enone-YTX (compounds #6/7/8 in [37]) with a molecular weight of 1048 Da. These two groups of isomers cannot be further resolved by mass spectrometry, and due to the lack of standard compounds, unambiguous assignment to one of the three alternative isomeric structures is not possible. The remaining seven YTX variants include a structurally unassigned YTX variant (entry 20/21 in [37]) with a molecular weight of 1062 Da, 9-methyl-41a-homo YTX (#10, 1170 Da), 44,55-dihydroxy-YTX (#13, 1176 Da), compound #15 ([37]; 1204 Da), compound #16 ([37]; 1086 Da), entry 45 ([37]; 1160 Da), and 41a-homo-44-oxotrinor YTX (#3 in [38]; 1132 Da).

Of the YTX group, YTX was present in levels up to 40 ng NT^−1^ in the 20–50 µm fraction, and up to 22 ng NT^−1^ in the 50–200 µm fraction (Supplementary Material Tables S6 and S7). YTX was detected in 66% of all stations in the 20–50 µm fraction, but only in 14% of the 50–200 µm fraction. The YTX variant with the highest record was #6/7/8 with 17 ng NT^−1^ in the 20–50 µm fraction of station 32 and 140 ng NT^−1^ in the 50–200 µm fraction of station 31. Compound #15 was also present with relatively high abundances of 51 ng NT^−1^ in the 20–50 µm fraction of station 40, and 68 ng NT^−1^ in the 50–200 µm fraction of station 31. All other YTX variants were detected at lower levels (Supplementary Material Tables S6 and S7). Interestingly, YTX entry 21/22 and #3 were only detected in the 20–50 µm size fraction, not the 50–200 µm fraction; the opposite was the case for YTX #10 and #16, which were only present in the 50–200 µm fraction, not the 20–50 µm fraction. It is also noteworthy that most YTX variants were predominantly present in the southern stations (23–41) of the cruise transect (Figure 2 and Figure 3).

### 2.6. Identified Potentially Toxic Plankton Species and Correspondence with Detected Phycotoxins

Integration of the results derived by morphological and metabarcoding approaches allowed for the detection of a total number of 20 potentially toxic species (Table 1).

The comparison of toxic microalgal taxa identified by metabarcoding and light microscopy revealed that seven species were detected using both approaches. Some species were discriminated only in the NGS dataset (e.g., species belonging to genera *Alexandrium* and *Pseudo-nitzschia*, as well as *Karlodinium veneficum, Polykrikos hartmannii*, and *Aureococcus anophagefferens*), whereas microscopy-based analysis reported two *Dinophysis* species (*Dinophysis sacculus* and *Dinophysis caudata*) that were not clearly reflected in the NGS dataset (instead, two OTUs sharing similarities with multiple *Dinophysis* species were registered). In terms of frequency distribution, most of the toxic species were more frequent in the LM analyses. Spearman rank correlation analyses between cell abundance and number of reads per sample of taxa identified using both methods revealed a statistically significant positive correlation only for *D. acuta* (r_s_ = 0.4), *L. polyedra* (r_s_ = 0.74), and *P. reticulatum* (r_s_ = 0.43).

**Figure 4:**
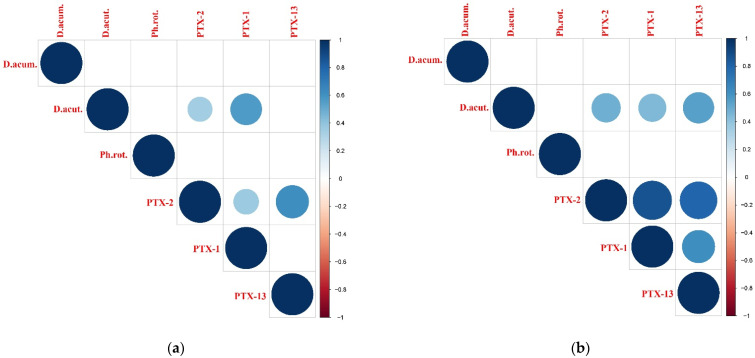

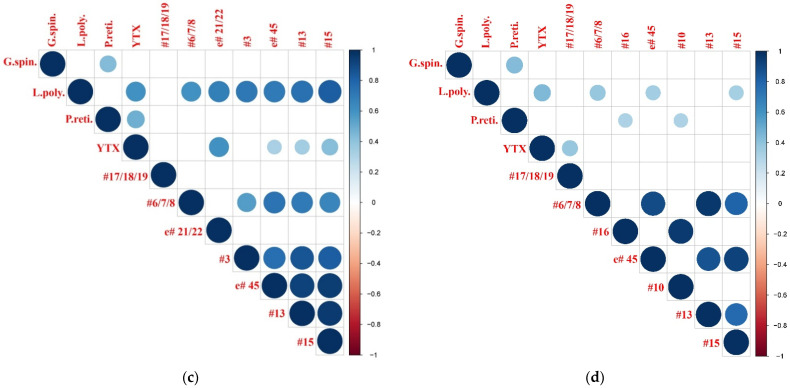
Correlation analysis between the toxic dinoflagellate species and the relevant toxin variants by fractions: (**a**,**c**) 20−50 µm fraction; (**b**,**d**) 50−200 µm fraction. Circle size and color intensity are proportional to the Spearman’s rho correlation coefficients. Empty spaces denote non-significant correlation (two-tailed *p*-value > 0.05). *Dinophysis acuminata* (D.acum.), *Dinophysis acuta* (D.acut.), *Phalacroma rotundatum* (Ph.rot.), *Gonyaulax spinifera* (G.spin.), *Lingulodinium polyedra* (L.poly.), *Protoceratium reticulatum* (P.reti.). *a,b *Dinophysis caudata* and *D. sacculus* were excluded from the analysis because they were detected at a limited number of stations. Toxin variant abbreviations are listed in Tables S6 and S7.

Spearman rank correlation analyses between plankton data (LM-based cell abundance and NGS-derived number of reads) and toxin abundances revealed that LM data correlated significantly with the toxin abundances (20–50 µm and 50–200 µm fractions), whereas NGS data showed only weak correlations (data not shown). The LM dataset was selected for further statistical analyses. Pectenotoxins were positively correlated only with *D. acuta* cell abundance (Figure 4a,b). In fraction 20–50 µm, the correlation with PTX-2 was weak, and that with PTX-1 was moderate (Figure 4a), whereas in the larger fraction (50–200 µm), all PTXs were moderately correlated with *D. acuta* cell abundance (Figure 4b). For yessotoxins, in the smaller fraction (20–50 µm), strong correlations (r_s_ values between 0.6 and 0.83) were found between *L. polyedra* cell abundance and all YTX variants (except for #17/18/19, which was not correlated with any of the possible identified producers), whereas *P. reticulatum* was moderately correlated with YTX only (Figure 4c). In the larger fraction (50–200 µm), only weak correlations were observed between *L. polyedra* and variants #6/7/8, e#45, and #15, and between *P. reticulatum* and variants #16 and #10, with the exception of YTX, which was moderately correlated with *L. polyedra* cell abundance (Figure 4d).

Detrended canonical analysis (DCA) of gradient lengths for community composition produced values much lower than 3 and close to 3 (DCA1 = 2.68, DCA2 = 2.99) for the abundances of toxin variants, and multicollinearity diagnostics of environmental data showed VIFs <5 for all explanatory variables. The resultant models explained nearly 50% of the variation in cell abundance data, and approximately 30% in the abundances of toxin variants (Table 2).

**Figure 5:**
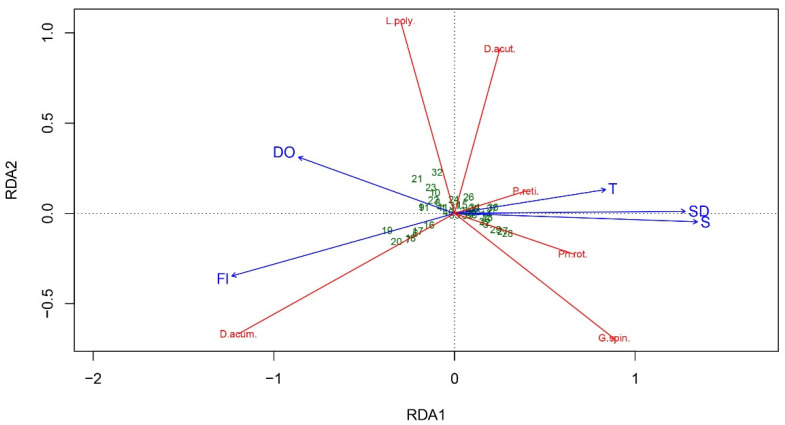
RDA correlation triplot (scaling type 1—lc scores—angles between vectors of response variables and explanatory variables reflect linear correlation) between the environmental variables and cell abundance data with fitted site scores. (T—temperature, S—salinity, Fl—fluorescence, DO—dissolved oxygen, SD—water transparency; D.acum.—*Dinophysis acuminata*, D.acut.—*Dinophysis acuta*, Ph.rot.—*Phalacroma rotundatum*, G.spin.—*Gonyaulax spinifera*, L.poly.—*Lingulodinium polyedra*, P.reti.—*Protoceratium reticulatum*). The green numbers represent stations.

RDA analysis showed that the first two axes explain 45% (unbiased variance) of the total variance of species cell abundance data, and therefore, the major trends have been captured by the model [39]. The triplot (Figure 5) shows the positive correlation of *D. acuminata* cell abundance with fluorescence and dissolved oxygen, as well as the positive correlations of *P. reticulatum*, *P. rotundatum*, *D. acuta*, and *G. spinifera* cell abundance with temperature and salinity. However, the short gradients of *P. rotundatum* and *P. reticulatum* rather indicate that they were present at most sampling sites or were related to intermediate ecological conditions [39]. *Lingulodinium polyedra* cell abundance variations had a weak positive correlation with dissolved oxygen.

**Figure 6:**
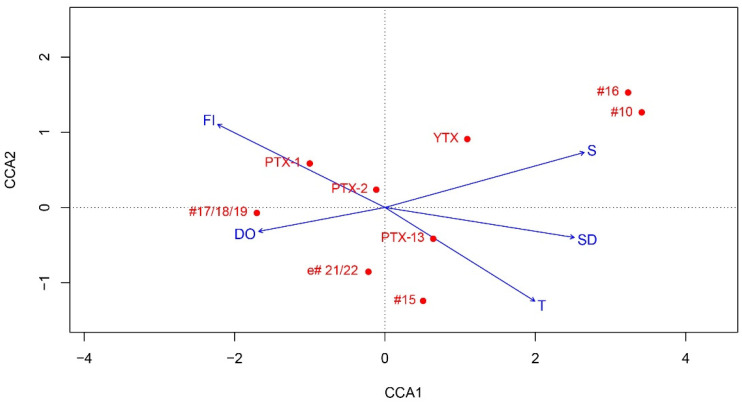
CCA biplot—(scaling type 1—lc scores)—toxin variant concentration scores constrained to environmental gradients (in situ environmental explanatory dataset, and toxin concentration response matrix). T, temperature; S, salinity; Fl, fluorescence; DO, dissolved oxygen; SD, water transparency. Toxin variant abbreviations are listed in Tables S6 and S7).

CCA analysis explained 36% of the total variance in toxins abundance data with the first two axes explaining 32%. The relative abundance of PTX-1 were associated with mid to high levels of FL and mid to low values of S, T and SD; PTX-2 abundances were linked to mid values of S, T, and SD and mid levels of FL; #17,18,19 were associated with mid to high FL and low T, SD and S; #16 with environment with high S, SD and T, and low FL; YTX, #15, e#45, #3, #6,7,8, #13 and PTX-13 with low S, T and SD and mid to low FL (Figure 6).

## 3. Discussion

The present study provides new high-resolution data regarding the presence and distribution of toxic microalgal species and associated phycotoxins in the northwestern Black Sea, combining molecular and morphological approaches for species identification with phycotoxin analyses. The application of different methods increased the detection power of toxic microalgae, and the integrated dataset allowed a better interpretation of the results, considering the strengths and limits of both methods [40]. In addition, correspondence between the occurrence of some species and related phycotoxins sheds light on possible toxin producers. Furthermore, some environmental drivers were found to have an effect on toxic plankton abundance and the distribution of toxin variants.

### 3.1. Pseudo-nitzschia and DA

Species of *Pseudo-nitzschia* are common members of the phytoplankton community in the Black Sea, often proliferating to bloom outbreaks [41]. In the present study, blooms of *Pseudo-nitzschia* spp. were observed at numerous stations, but no DA was detected in the corresponding samples. Cells categorized into the *Pseudo-nitzschia delicatissima* group were much more abundant than those of the *P. seriata* group, consistent with previous reports in Bulgarian and Romanian waters [19]. Commonly, both groups comprise multiple species that frequently co-occur [42]. Presently, nine *Pseudo-nitzschia* species have been reported in the Black Sea, with six of them generally reported as capable of DA production [41]. In the current study, according to NGS results, three potentially toxic species were discriminated, *P. calliantha*, *P. delicatissima*, and *P. pungens*, with the first one being dominant within the samples, in agreement with previous studies in Bulgarian coastal waters [43]. *Pseudo-nitzschia calliantha* is the only *Pseudo-nitzschia* species in the Black Sea proven to be toxigenic, with a maximum DA cell quota of 0.95 pg cell^−1^ [26], which is comparable to strains from other regions [44]. Globally, both toxic and non-toxic strains of the three detected *Pseudo-nitzschia* species have been documented [45]. The relationships between DA production and environmental factors are complex and sometimes controversial [46]. Salinity is among the multiple factors that affect DA production, and a significant decline in DA production has been registered at lower salinities (<20) both in natural populations [47] and laboratory cultures [48]. The Black Sea is the world’s largest brackish water body, and the average salinities of water column during the sampling campaign were between 16.6 and 18.7 (Supplementary Material Table S1). The low salinity could be among the leading factors affecting the low levels of DA registered in the Black Sea [24] and the lack of ASP events in that region. This hypothesis is supported by the absence of DA in the brackish Baltic Sea, although numerous *Pseudo-nitzschia* species have been registered there [49], sometimes forming blooms [50,51]. However, it is unwise to draw general conclusions, considering that various factors (abiotic and biotic) are reported to control the toxin production [44,45,52]. In addition, the absence of DA in the planktonic samples of the current survey could also be attributed to the final phase of the bloom and predominantly disintegrated *Pseudo-nitzschia* cells, and accordingly, the release of cellular domoic acid into the water [53,54]. Nevertheless, earlier reports on domoic acid measured in samples from the Black Sea provide confirmation of the potential of local *Pseudo-nitzschia* strains to produce this phycotoxin, and further investigations are vital to elucidate the effects of different factors on their growth and toxicity at a regional level.

### 3.2. Alexandrium, Gymnodinium catenatum, and PSP

DNA sequence data indicate the presence of several potential PSP-toxin-producing species in the plankton samples of this survey: *Alexandrium andersonii*, *A. ostenfeldii*, *A. minutum*, and *Gymnodinium catenatum*. In addition, more *Alexandrium* species remained genetically unidentified due to the high similarity in the target region between two different toxic *Alexandrium* species. Our LM analyses could not identify *Alexandrium* at the species level, as identification is tedious and requires careful dissection and/or fluorescence staining of thecal plates. Nevertheless, variability in cell morphology and size was observed to be in line with molecular data, providing evidence for the co-occurrence of several species (Supplementary Material Tables S2 and S3). In any case, no PSP toxins were detected in the plankton samples. This may be due to low cell numbers and the relatively high detection limit (LOD) of PSP toxins. The individual LODs of PSP toxins are highly variable, and range from 4 ng NT^−1^ for dcGTX-3 to 106 ng NT^−1^ for GTX-1 (Supplementary Material Table S5). This means that depending on the toxin composition of a certain species, the LOD may vary by more than one order of magnitude. Assuming a PSP toxin cell quota of 5 pg cell^−1^ and given an LOD of 5–100 ng NT^−1^, approximately 1000 to 20,000 cells NT^−1^ would be necessary in order to detect PSP toxins in plankton samples. The highest abundance of *Alexandrium* cells was 242,000 cell NT^−1^ at station 18 (Supplementary Material Table S2), which is above the estimated detection threshold. However, this estimation relies on some uncertainties, and, importantly, non-toxic *A. margalefii* was also present in the samples. PSP toxins have been registered in the Black Sea in farmed and wild mussels at low levels [25,30,33] in concomitant presence of *Alexandrium* cells; however, no PSTs were detected in the plankton samples [33]. Numerous *Alexandrium* species have been reported in the Black Sea on the basis of morphological [55] and molecular data [22,56], but high densities of *Alexandrium* spp. rarely occur in the Black Sea, although occasional blooms of *A. monilatum* and *A. ostenfeldii* have been observed [16,57]. It is worth noting that *A. ostenfeldii* form dense blooms and produce PSP toxins in brackish areas of the Baltic Sea [58,59] and of the Netherlands [60], revealing the toxigenic nature of strains adapted to low salinity.

The sequence signature of another potential PSP producer, *Gymnodinium catenatum*, was detected in 12 samples, mainly along the Bulgarian coast. This species has been previously identified in the Black Sea on the basis of 18S rRNA gene sequencing in the water column [56] and in sediment [22,61]. The species is considered to have been introduced after the year 2000 [62]. However, no strains or morphological confirmation of its presence, or any toxinological data of Black Sea populations, are available, which might be due to general difficulties in the identification of species of *Gymnodinium*, and thus there is limited information on its distribution and abundance.

### 3.3. Dinophysis, Phalacroma rotundatum, and OA/DTX/PTX Distribution

The genus *Dinophysis* was identified in all samples in this study, with *D. acuta* and *D. acuminata* being dominant. In addition, *P. rotundatum* was found at most of the studied areas. Among the DSTs, only PTXs were detected and quantified at all sampling stations. PTX-2 was the major toxin, reaching levels much higher (up to 206 ng NT^−1^ in the 20–50 µm size fraction and up to 426 ng NT^−1^ in the 50–200 µm size fraction) than previously reported in plankton samples from the Bulgarian coast (0.862 ng PTX-2 NT^−1^) [24], and also from other basins (26 ng NT^−1^ in Ambon Bay, Indonesia [63]; 43 ng NT^−1^ in Southeastern Pacific fjords [64]). PTX-2 and PTX-2sa also dominated in farmed mussels from Russian waters, with a corresponding presence of *D. caudata* and *P. rotundatum*, while okadaic acid (OA) and the related congener (DTX-1) were below the level of quantification [28]. No correlation between *P. rotundatum* cell abundance and PTXs was observed in the current study; this is in accordance with recent data indicating that this heterotrophic species may be toxin vector but not a de novo toxin producer [65]. It has been reported that the toxin profile of *Dinophysis* spp. is strain/region-specific [66]. PTX-2 is the dominant toxin related to *D. acuminata*, *D. acuta*, *D. caudata*, and *D. sacculus* from different locations [67,68,69,70,71,72,73,74]. In the current investigation, PTX-2 levels were positively correlated with *D. acuta* cell abundance for both fractions (20–50 μm, weak correlation; 50–200 μm, moderate correlation). In addition, *D. acuta* was also correlated (moderately for both fractions) with PTX-1 and PTX-13, toxins that have not been reported previously in the Black Sea. Globally, there are fewer records of PTX-1 and PTX-13 in plankton samples compared to PTX-2. PTX-1 was reported for the first time in plankton samples of the North Sea (at levels comparable with those quantified in the Black Sea), correlated with *D. acuminata* cell abundance [75], whereas PTX-13 was first isolated from extracts of *D. acuta* from New Zealand [76]. Despite the observed association between PTXs and *D. acuta*, it is difficult to confidently determine whether this was the only source of toxins, considering the weak to moderate correlation. The cell size range (Supplementary Material Table S2) suggests that most of the cells should be retained in the larger fraction (50–200 μm). Furthermore, the data indicate that all identified *Dinophysis* species potentially produce PTXs, and that there is high variability in the toxin cell quota of *Dinophysis* strains and/or species [66].

*Dinophysis* spp. commonly occur in the Black Sea phytoplankton community under a wide temperature and salinity range, not usually reaching high abundance [77,78,79]. Nevertheless, DSTs are so potent that they may cause harm even at low cell densities [80]. Globally, *Dinophysis* bloom initiation and toxin production were associated with various environmental parameters, e.g., stratification, temperature, salinity, irradiance, upwelling, nutrients, or dissolved oxygen [63,81,82,83,84]. Vershinin and Kamnev [27] suggested a positive correlation between water temperature and *Dinophysis* development and consequent DSP cases in the Black Sea. Similarly, Peteva et al. [24] also reported an increase in *Dinophysis* cell abundance and PTXs with increasing spring temperatures; on the other hand, no pectenotoxins were detected in summer [25]. Consistently, in the current research, *D. acuta* cell abundance was related to temperature (Figure 5). Pectenotoxins were associated with mid to low values of T (Figure 6), in agreement with laboratory studies showing an influence of temperature on the cellular production of PTX-2, and higher cellular content of PTX-2 at lower temperatures [85].

Pectenotoxins were quantified in plankton samples at all sampling stations; however, they have very low oral toxicity and pose negligible risk to humans [86]. In addition, the reported levels of DSTs in mussel samples from the Black Sea were far below the regulatory limit [24,25]. Therefore, the risk of significant DSP toxin outbreaks in the northwestern Black Sea is low, as also reported by other authors [87].

### 3.4. Protoceratium reticulatum, Lingulodinium polyedra, Gonyaulax spinifera, and YTX Profiles

The potential YTX-producing species *Protoceratium reticulatum*, *Lingulodinium polyedra*, and *Gonyaulax spinifera*, detected in most of the samples in the current study, are common components of the plankton community along the Bulgarian Black Sea, although not reaching high abundances [79]. Yessotoxins were quantified at many stations, and numerous YTX variants were reported for the first time for the Black Sea. YTX was the major toxin variant, but was detected at levels much lower (up to 40 ng NT^−1^) than those reported in San Jorge Gulf, Argentina (8040 ng NT^−1^) [88]. Yessotoxins, tentatively related to *Lingulodinium polyedra* and *Gonyaulax spinifera*, have been detected earlier from Black Sea mussel samples [24,25,29,87], but toxins have not been found in plankton [24]. Strong relationships were found between *Lingulodinium polyedra* cell numbers and all of the detected YTX variants (except #17/18/19). *Protoceratium reticulatum* was moderately associated with YTX, which has been reported as the major toxin of the species [89], whereas *G. spinifera* showed no correlation with any YTX variant. Correlations were found mainly for the 20–50 µm size fraction, in accordance with the cell size of both species (Supplementary Material Table S2). Various strains from different regions of *L. polyedra* and *P. reticulatum* showed high variability in YTX levels and toxin profiles, which may also dependent on environmental factors [90,91,92,93,94,95]. In the current study, YTX variants showed distinct relationships with environmental variables (temperatures, salinity, water transparency, and fluorescence) (Figure 6), suggesting an effect on the toxin profile in the Black Sea. For example, some of the YTX analogues were associated with salinity, in accordance with studies in the Skagerrak coast of Sweden, where change in proportion of the yessotoxin analogues with decreasing salinity was reported, and a positive correlation between cellular toxin content of *L. polyedra* and salinity was observed [94].

The low levels of YTXs in plankton and previously measured in mussel samples [24,25,87], and the reported low oral toxicity of YTX analogues [96,97], suggest a negligible risk of acute intoxication in the western part of the Black Sea. On the other hand, the persistent presence of yessotoxins could result in chronic exposure from shellfish consumption, which might pose a threat to human health [98].

### 3.5. Amphidomataceae and AZA

Among the members of the marine dinoflagellate family Amphidomataceae, some species of *Azadinium* and *Amphidoma languida* produce azaspiracids [99,100,101]. In the present study, *Amphidoma languida* was the only toxic member of the family registered in the NGS results, represented with few sequences at just three stations. In addition, non-toxigenic *Azadinium trinitatum* was detected at one station (data not shown). qPCR analysis also confirmed the presence of Amphidomataceae in some samples (11 stations), but the targeted toxic species *Azadinium spinosum*, *Azadinium poporum*, and *Amphidoma languida* were not detected. There was an agreement for detecting Amphidomataceae by both methods for three stations, whereas the other stations differed. The observed discrepancy could be mainly attributed to the different sampling approaches used for the two methods (plankton net sample for NGS vs. CTD water sample for qPCR). Considering the small cell size (<20 µm) of most Amphidomataceae species [101], net samples (mesh size 20 µm) in general might not be well suited for targeted studies on this group of microalgae. However, the net haul over ~ 20 m covered a relatively large range of the water column. In contrast, species-specific qPCR analysis of selected sampling depths indicated the lack of toxic Amphidomataceae species, which is in accordance with the lack of azaspiracids in CTD samples. A relatively low number of OTUs in NGS analysis, together with the lack of signals in qPCR and AZA analysis, as well as no observation by microscopy reveal rather background abundances of toxigenic Amphidomataceae in the samples. Overall, the data shown here represent first results, and further studies with a focus on these relatively small-sized species and their toxins are needed to fully evaluate the biogeography of Amphidomataceae in the Black Sea.

### 3.6. Karlodinium veneficum and KmTx

The small marine dinoflagellate *Karlodinium veneficum* has been known as a notorious producer of karlotoxins (KmTx) [102,103,104] with an increasingly high number of KmTx variants. However, in contrast to AZA, which have a low LOD, KmTx are not detected with high sensitivity, and have an average LOD of 200 pg L^−1^. Considering that Mediterranean strains of *K. veneficum* have an individual KmTx cell quota of 200 fg cell^−1^ [104], KmTx theoretically should be detectable above a threshold of approximately 100,000 *K. veneficum* cells L^−1^ based on a water sample of 2 L. According to NGS data (Supplementary Material Table S3), *K. veneficum* was among the dominant potentially toxic dinoflagellate species present during this survey. However, metabarcoding only provides data in terms of relative abundance, and the number of sequences cannot be directly used as a proxy for actual cell abundance [105,106]. Unfortunately, the difficult morphological identification of *Karlodinium*, even at the genus level, under LM in a formalin-fixed sample also did not allow the quantitative assessment of this group of organisms in the samples. Regardless, no KmTx was detected at any station. There are several explanations for this: On one hand, there are reports of Mediterranean strains of *K. veneficum* with lower KmTx cell quotas than 200 fg cell^−1^ [104]. On the other hand, a high degree of toxin variation has been observed in different ecotypes among strains [102,103], and the chemical variability of KmTx is not yet fully explored. It may very well be the case that yet unknown KmTx might be produced by Black Sea *K. veneficum* populations that would not have been detected by the targeted LC-MS/MS approach used in this study. An important issue is the lower variability of the 18S rRNA region, and the limited SSU rDNA sequence data for different *Karlodinium* species in GenBank, which may lead to molecular misidentification considering that, until now, *K. veneficum* was reported in the Black Sea based only on 18S rRNA gene metabarcoding [21,56]. Further studies are necessary for elucidating the taxonomic identity of *Karlodinium* species in the Black Sea and whether it is capable of karlotoxin production.

### 3.7. Other Potentially Toxic Species Identified in the Study

Three other potentially toxic species were detected during the current survey.

*Prorocentrum cordatum* is a common species in the Black Sea, often proliferating to bloom abundances in the past [19]. Model data have shown that there is a high risk of *P. cordatum* mass outbreaks for about 16% of the whole Black Sea area [107]. Black Sea strains of *P. cordatum* did not show toxic potential in situ [108], as well as in laboratory conditions after the application of mouse bioassay [109].

*Pheopolykrikos hartmannii* was previously identified, sporadically, in plankton and sediment samples from the Black Sea [21,40]. In the current study, it was detected in more than half of the samples only with the molecular approach. No blooms have been reported regionally, although bloom densities and ichthyotoxicity were documented in a lagoonal brackish system [110].

*Aureococcus anophagefferens* is a picoplanktonic member of the Pelagophyceae that causes harmful brown tides in estuarine waters [111]. It was identified earlier in the Black Sea only via eDNA metabarcoding [56], and data on its distribution are very limited.

## 4. Conclusions

The integrated approach, applied in this study for the first time, allowed better insight into the composition and distribution of toxic microalgal species and phycotoxins in the Black Sea. Considering the methodological constrains of the individual methods, our results highlight the significance of the combined data for a better understanding of the current plankton–phycotoxin variability pattern in the NW Black Sea. Numerous toxic microalgae and phycotoxins persistently occur in the western part of the Black Sea. PTX-1 and PTX-13, as well as some YTX variants, were recorded for the first time in the basin. A positive correlation was found between the abundance of *D. acuta* and PTXs, and between *L. polyedra* and *P. reticulatum* and YTXs. However, culture studies are required for the elucidation of the exact toxin profiles of Black Sea strains. Application of more sophisticated morphological methods (e.g., SEM) and multiple DNA markers will increase the identification power in decoding the microalgal diversity. In addition, the effect of the environmental variables on the toxic microalgae and toxicity needs to be more thoroughly explored (covering more key factors, e.g., nutrients, pH) considering the observed complex associations. The low levels of the identified phycotoxins and their low oral toxicity does not exclude the threat of chronic toxic exposure.

## 5. Materials and Methods

### 5.1. Study Area and Sampling

**Figure 7:**
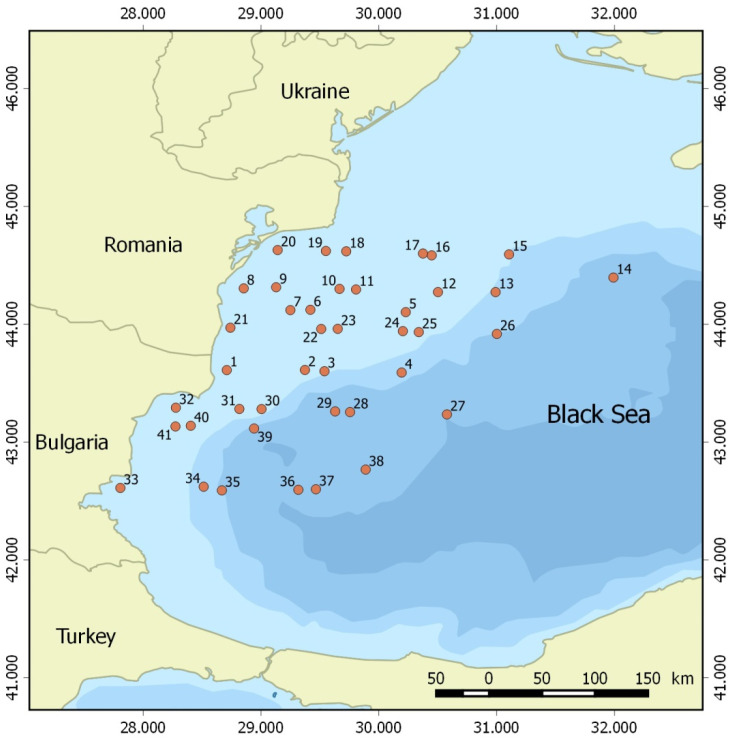
Study area and sampling stations.

Data were collected during the bio-optical oceanographic cruise (Project “BLACK SEA COLOR”, Contract № 4000123951/18/NL/SC, Plan for European Cooperating States) carried out from 15th May 2019 to 4th June 2019 aboard RV Akademik in the northwestern Black Sea (Romanian and Bulgarian waters). In total, 41 stations were sampled (Figure 7; Supplementary Material Table S1). CTD profiles were recorded at each station using an SBE-911 CTD system outfitted with a fluorescence sensor. Water transparency was measured in situ at each station using a Secchi disk. Parameters of seawater, i.e., temperature (T), salinity (S), fluorescence (Fl), and dissolved oxygen (DO), from the CTD readings were analyzed at each station for the surface (1 m), the bottom of the sampling layer, and average for the sampled water column (Supplementary Material Table S1).

### 5.2. Plankton Sampling

At each station, one vertical net tow was taken from the water column using a 20 μm plankton net (438-030, Hydro-Bios, Kiel, Germany). The depth of the hauls was fixed to 0–20 m, with the exception of some stations (Supplementary Material Table S1). The collected net tow concentrates were adjusted to 1 L with filtered seawater. In total, 50 mL aliquots were fixed with formaldehyde solution (4% final concentration), buffered to pH 8–8.2 with disodiumtetraborate for microscopic analyses, and for DNA (metabarcoding) analysis, 150 mL aliquots were filtered under gentle vacuum (<0.2 bar) through 1 μm pore-size polycarbonate filters (Whatman, USA). The filters were immediately frozen and stored in liquid nitrogen until further analysis. The remaining 800 mL of each net haul concentrate were fractionated over a sieve array, consisting of 200 µm, 50 µm, and 20 µm Nitex sieves. The three fractions were each transferred to a 50 mL centrifuge tube and adjusted to 30 mL with filtered water. The contents of each tube were separated into two aliquots for the analysis of lipophilic toxins (including domoic acid) as well as for the analysis of the hydrophilic PSP toxins. Prior to aliquotation, homogenization of the samples was ensured, and the rapidly sedimenting material was brought back into suspension by shaking. Subsequently, the aliquots were centrifuged at 5289× g for 20 min (PK130, JOUAN Italia Srl, Rodano, MI, Italy) and the supernatant was decanted. In some cases, after partial removal of supernatant, further centrifugation at 5289× g for 10 min was necessary to pellet residual suspended solids from the remaining supernatant. After decanting again, the pellets were resuspended and quantitatively transferred to a 2 mL cryovial each. Samples were centrifuged one last time at 16,163× g for 15 min (Sigma 1–14, Osterode, Germany), and the supernatants were removed and the pellets stored at −20 °C until analysis.

In addition to net tows, plankton samples were also collected at each station by Niskin bottles at up to three different depths (Supplementary Material Table S4). From each depth, three liters were pre-screened through a 20 µm Nitex sieve and subsequently pooled. In duplicate, 0.6–4.0 L (depending on the particle content) of the mixture was filtered under gentle vacuum (<200 mbar) through 3 µm Whatman polycarbonate filters (Ø 47 mm, GE Healthcare, Little Chalfont, UK). One filter was used for DNA extraction and qPCR analyses, and the other filter was used for azaspiracid (AZA) analysis. The filters for DNA were stored at -20 °C until extraction, whereas the still wet filters for AZA analysis were attached to the inner wall of a 50 mL centrifuge tube. The filters were then carefully rinsed with one mL methanol (99.4%, Marvin Ltd., Plovdiv, Bulgaria). The methanol accumulating in the bottom tip of the tube was repeatedly used to rinse the filter until complete decolorization. The methanol extract was transferred to a 2 mL cryovial and stored at −20 °C until extraction.

### 5.3. Microscopy

Taxonomic identification and cell counts were done under inverted microscope (Nikon Eclipse TE2000-U) connected to a video-interactive image analysis system (L.U.C.I.A, Version 4.8, Laboratory Imaging Ltd., Prague, Czech Republic) at 400× magnification in Sedgwick-Rafter counting chambers. A total of 400 cells were counted from each sample, and rare and large species were checked in the whole counting chamber [112]. Cell abundance was expressed as cells per net tow (cells NT^−1^). Taxonomic nomenclature was in accordance with the online database of World Register of Marine Species (WoRMS) http://www.marinespecies.org/, accessed on 6 October 2021. Due to the impossible taxonomic identification at the species level under LM, *Pseudo-nitzschia* spp. were separated into two groups on the basis of cell width: (1) seriata group (>3 µm width) and (2) delicatissima group (<3 µm width) [113], whereas *Alexandrium* cells were pooled as *Alexandrium* spp. The dimensions of at least 10 cells from each potentially toxic species were measured for each sample. The QC/QA (quality assurance/quality control) of the data was performed following the quality control guidelines for phytoplankton [114].

### 5.4. DNA (metabarcoding) Analysis

The frozen filters were thawed and genomic DNA were extracted using a DNeasy PowerWater Kit (QIAGEN) according to the manufacturer’s instructions. The DNA samples were stored at −20 °C until further processing. For detection of eukaryotic species, universal primers for the 18S rRNA gene V7–V9 variable region (18S-V7F: TGGAGYGATHTGTCTGGTTDATTCCG and 18S-V9R: TCACCTACGGAWACCTTGTTACG; modified from Tanabe et al. [115]) were used. The construction of paired-end libraries and HTS on Illumina Miseq 300 PE platform (Illumina, San Diego, CA, USA) were performed by Macrogen Inc. (Seoul, South Korea). One of the samples (st.14) failed in library construction, so finally, 40 samples in total were sequenced. The procedures and techniques, applicable to the treatment of the obtained sequences, selection, and taxonomic identification of operational taxonomic units (OTUs), were administered according to the workflow described in Dzhembekova et al. [43] with the exception that sequences with length > 300 bp were truncated to 300 bp by trimming the 3′ tails. The trimmed sequences shorter than 250 bp were filtered out. Taxonomic assignment was performed using BLAST against a sequence database downloaded from GenBank. Sequences were clustered to OTUs at ≥99.1% similarity level. When considering the taxonomic identification, a reference similarity threshold ≥99% was set for identification at the species level. IOC-UNESCO Taxonomic Reference List of Harmful Micro Algae [116] was used as a reference database of toxic microalgal species selection both for microscopy and NGS data. For additional verification, representative sequences of all OTUs associated with toxic species were also manually BLAST searched from the GenBank online database [117]. DNA sequences for this study can be found in the DDBJ Sequence Read Archive under accession number DRA014629 (biosamples SAMD00515557, SAMD00515558, SAMD00515560, SAMD00515561, SAMD00515563, SAMD00515564, SAMD00515566–SAMD00515574, SAMD00515576, SAMD00515577, SAMD00515579–SAMD00515586, SAMD00515588, SAMD00515589, SAMD00515592, SAMD00515593, SAMD00515596, SAMD00515598, SAMD00515599, SAMD00515602, SAMD00515603, SAMD00515606, SAMD00515607, SAMD00515610, SAMD00515611, SAMD00515615, SAMD00515616).

### 5.5. DNA Extraction and qPCR

Extraction of DNA from the filtered CTD samples was carried out by transferring the filters to a glass petri dish (Brand, Wertheim, Germany) and adding 500 µL the SL1 lysis buffer, provided by the NucleoSpin Soil DNA extraction kit (Macherey & Nagel, Düren, Germany). The filtered material was scratched off the filters using cell scrapers (16 cm, Sarstedt, Nümbrecht, Germany) and transferred with the buffer to the beat tubes provided by the DNA extraction kit. The extraction was performed according to the manufacturer´s instructions, with a slight modification. The beat tubes were not vortexed but shaken in cell disrupter (FastPrep FP120, Thermo-Savant, Illkirch, France) for 45 s initially and then for another 30 s, at a speed of 4.0 m s^−1^ each. For final DNA elution, two elution procedures with 50 μL of the provided buffer each (to a final elution volume of 100 μL) were carried out to maximize the overall DNA yield. DNA samples were stored at −20 °C until further analysis.

DNA samples were screened by qPCR on the presence of Amphidomataceae. A family-specific SYBR Green qPCR assay introduced by Smith et al. [118] was used to detect overall amphidomatacean DNA in the samples. Reactions and cycler conditions were set as described in Tillmann et al. [119]. All reactions were performed in duplicate per sample, and runs contained positive controls (*Az. poporum*, strain UTH-D4; 1 ng µL^−1^), negative controls (*Alexandrium* spp.; 1 ng µL^−1^), and non-template controls (NTC; high-grade, nuclease-free water). Samples were considered as being positive, if at least one of the replicates showed a fluorescence signal above the threshold before cycle 37. Assay performance was also evaluated by melt curve analysis for every single reaction. In addition, positive evaluated samples for amplification and melt temperature were subsequently run on an agarose gel (1%, 70 mV, 30 min) in TE buffer to verify correct amplicon length (179 bp).

The positive amphidomatacean samples revealed by the SYBR Green assay were quantitatively tested with the species-specific TaqMan qPCR assays on AZA-producing species *Az. spinosum*, *Az. poporum* and *Am. languida* [120,121], and were performed as described in Wietkamp et al. [122]. DNA standard curves were included as 10-fold dilution series of target species DNA (10 ng μL^−1^ to 100 fg μL^−1^) from exponentially growing cultures of *Az. spinosum* (strain 3D9), *Az. poporum* (strain UTH-D4) and *Am. languida* (strain AND-0920).

The limit of detection (LOD) and limit of quantification (LOQ) were defined following Forootan et al. [123]. The LOD was assigned to the lowest standard curve concentration, for which all three replicates showed amplification, but values were outside the 95% confidence interval of the standard curve. The LOQ was defined as the lowest standard curve concentration, for which all three replicates showed amplification within the 95% confidence interval. For the standard curves of all three qPCR assays on the field samples, the standard curve resolution applied did not allow differentiation between LOD and LOQ, which were both 0.1 pg μL^−1^. Taking the filter and extraction volume into account, the LOD of 0.1 pg µL^−1^ in the species-specific assays corresponded to 1–6 cells L^−1^ filter volume.

### 5.6. Toxin Extraction

For the extraction of PSP and lipophilic toxins (including domoic acid), 400 µL 0.03 M acetic acid (p.a., Merck, Darmstadt, Germany) for PSP toxins and 400 µL methanol (HPLC-grade, Merck) for lipophilic toxins were added to the respective cell pellets. To each sample, 0.9 g of Lysing Matrix D (Thermo Savant, Illkirch, France) were added as well. After sealing and vortexing the cryovials, cells were lysed by reciprocal shaking at maximum speed (6.5 m s^−1^) for 45 s in a Bio 101 FastPrep device (Thermo Savant). Subsequently, homogenates were centrifuged at 16,100× *g* and 10 °C for 5 min (5415R, Eppendorf, Hamburg, Germany). The extracts were transferred to centrifugation filters with a pore size of 0.45 µm (Millipore Ultrafree, Eschborn, Germany) and centrifuged at 16,100× *g* and 10 °C for 1 min. The filtrates were finally transferred to high performance liquid chromatography (HPLC) sample vials (2 mL, Agilent, Waldbronn, Germany) and vials for PSP toxin analysis were sealed with rubber crimp caps (Agilent), while vials for the analysis of lipophilic toxins were sealed with crimp caps (Agilent). If necessary, the extract was transferred to a cone-shaped HPLC sample vial (Vial, crimp top, micro sampling, Agilent Technologies) to increase the fill level. Samples were stored at −20 °C until measurement.

The methanolic extracts of bottle sample water filters were completely evaporated in a gentle stream of nitrogen. The dry samples were taken up in 300 µL acetone (HPLC-grade, Merck) and vortexed for 30 s. The extracts were transferred to a centrifugation filter with a pore size of 0.45 µm and centrifuged at 16,100× *g* and 10 °C for 1 min. The filtrates were finally transferred to a conical HPLC sample vial and sealed with a lid with a silicone septum.

### 5.7. LC-FLD and LC-MS/MS Analysis

Liquid chromatography with post-column derivatization and fluorescence detection (LC-FLD) was used for the determination of PSP toxins. The chromatograph (LC1100, Agilent) consisted of a G1379A degasser, a G1311A quaternary pump, a G1329A autosampler, a G1330B autosampler thermostat, a G1316A column thermostat, and a G1321A fluorescence detector. The system was coupled to a post-column derivatization unit (PCX 2500, Pickering Laboratories, Mountain View, CA, USA). Separation was performed on a Luna C18 RP column (Phenomenex, Aschaffenburg, Germany) with a length of 250 mm, an inner diameter of 4.6 mm and a particle diameter of 5 µm. A Phenomenex SecuriGuard was used as the precolumn. Instrumental details are given in supplementary information (Supplementary Material Tables S8 and S9). Data acquisition and processing was performed by the HP ChemStation software (Agilent). A PSP mixed standard was used to identify and quantify the toxins, which contained the following components: saxitoxin (STX), neosaxitoxin (NEO), decarbamoyl-saxitoxin (dcSTX), gonyautoxins 1–4 (GTX-1 to -4), decarbamoyl-gonyautoxin-2/3 (dcGTX2/3, B1 and C1/2). These individual standards were obtained from the Certified Reference Materials Programme of the Institute of Marine Biology, Halifax, NS, Canada.

For the measurement of lipophilic toxins, including domoic acid, ultraperformance liquid chromatography (UPLC®) coupled with tandem quadrupole mass spectrometry (LC-MS/MS) was used. The UPLC system included a column oven, an autosampler and a binary pump (AQUITY I UPLC Class, Waters, Eschborn, Germany). The separation was carried out on a RP-18 column (Purospher®STAR endcapped (2 µm) Hibar® HR 50-2.1 UPLC, Merck) equipped with a precolumn (0.5 µm, OPTSSOLV® EXP™, Sigma-Aldrich, Hamburg, Germany). This system was coupled to a triple quadrupole mass spectrometer (Xevo® TQ-XS, Waters). Data were acquired and analyzed with Masslynx (version 4.2, Waters). To uniquely identify the toxins, in addition to the mass transitions defined in the selected reaction monitoring (SRM) mode, the retention times of the toxins of the standards were compared with those in the samples. For quantification, an evaluation method was used which contained the specific transitions and default settings, except for the smoothing function, which was turned off. In some cases, enhanced production spectra were recorded. These were used for the identification of known substances by comparing the recorded characteristic fragmentation patterns with those in the literature. The various eluents and gradients used for the different toxin analyses are described in supplementary information (Supplementary Material Tables S10–S15). Certified standard solutions were used to identify and quantify toxins. These were gymnodimine A (GYM-A), 13-desmethylspirolide C (SPX-1), okadaic acid (OA), dinophysistoxin-1, and -2 (DTX-1, DTX-2), pectenotoxin 2 (PTX-2), yessotoxin (YTX), domoic acid (DA), and azaspiracid 1 (AZA-1). GYM-A, SPX-1, OA, and PTX-2, like the PSP standards, were obtained from the Institute of Marine Biology from Canada, while DTX-1, DTX-2, YTX, DA, and AZA-1 were obtained from the Laboratorio CIFGA S.A., Lugo, Spain. In addition, a goniodomin A (GD-A) standard was used, which was obtained from A. Andersen [124] and a KmTx-2 standard provided by A. Place [104].

### 5.8. Statistical Analysis

Spearman rank correlation was used to identify statistically significant species associations with toxin abundance by fractions. Detrended canonical analysis (DCA) was utilized for estimation of the gradient lengths to justify feasibility of linear or unimodal constrained ordination methods. Redundancy analysis (RDA) was applied on species data and canonical correspondence analysis (CCA) to toxin data, constraining species cell and toxin abundances by selected abiotic and biotic variables. The variance inflection factors (VIF) of the variables were used as a test for multicollinearity of data to avoid erroneous model interpretations. Analysis of variance (ANOVA) was used for the assessment of the RDA and CCA models’ statistical significance, RDA and CCA axes and models’ terms (explanatory variables) significance (α = 0.05). The above listed analyses and graphical representations were performed in Matlab [125] and R environment using the statistics and programming software R 4.1.2 [126], packages ‘hmisc’ [127] and ‘vegan’ [128], available through the CRAN repository [129].

### 5.9. Data Used for Statistical Analyses

Environmental parameters selected for analyses were taken as an average values over the same depth as the net tows plankton and toxin samples at each station for temperature (T), salinity (S), fluorescence (Fl), dissolved oxygen (DO) from the CTD readings, and water transparency (SD) (Supplementary Material Table S1). The fluorescence data used were expressed as relative values (mg m^−3^) not calibrated against chl a measurement. Cell abundance data only represent species classified as producers of detected toxins. Abundance and toxin data, along with the respective cumulative abundances of toxin variants (integrated values of fractions 20–50 µm and 50–200 µm) used for RDA and CCA, were gathered at all 41 sampling sites.

## Figures and Tables

**Table 1:** Integrated list of potentially toxic species identified by light microscopy (LM) and metabarcoding (NGS) (“+” identified; “-” not identified).

Species	LM	NGS
*Pseudo-nitzschia calliantha*	-	+
*Pseudo-nitzschia delicatissima*	-	+
*Pseudo-nitzschia pungens*	-	+
*Alexandrium andersonii*	-	+
*Alexandrium minutum*	-	+
*Alexandrium ostenfeldii*	-	+
*Amphidoma languida*	-	+
*Dinophysis acuminata*	+	+
*Dinophysis acuta*	+	+
*Dinophysis caudata*	+	-
*Dinophysis sacculus*	+	-
*Gonyaulax spinifera*	+	+
*Gymnodinium catenatum*	-	+
*Karlodinium veneficum*	-	+
*Lingulodinium polyedra*	+	+
*Phalacroma rotundatum*	+	+
*Polykrikos hartmannii*	-	+
*Prorocentrum cordatum*	+	+
*Protoceratium reticulatum*	+	+
*Aureococcus anophagefferens*	-	+

**Table 2:** Redundancy analysis (RDA) and canonical correspondence analysis (CCA) models: statistical significance outcomes and percentage contributions of explanatory variables to total variance explained by the models.

	RDA Model Cell Abundance vs. Environmental Data	CCA Model Toxin Variant Abundance vs. Environmental Data
*Proportion of the total variance (inertia) explained*		
Constrained	55.68%	36%
*Model significance (ANOVA)*		
*p*-values	*p* = 0.001	*p* = 0.001
R^2^	0.57	n/a
R^2^_adj_	0.49	n/a
*Axes significance (ANOVA) and contribution to total variance explained by the model*		
*p*-values	RDA_1_ axis *p* = 0.001; 36.04% RDA_2_ axis *p* = 0.001; 8.94% Contribution of variables to the axis explains the biggest portion of variance in data: RDA_1_ – T, S, DO, and SD	CCA_1_ axis *p* = 0.001; 22.92% CCA_2_ axis *p* = 0.048; 9.75%. Contribution of variables to the axis explains the biggest portion of variance in data: CCA_1_ – T, S, Fl, and SD
*Model term significance (ANOVA) and percentage contribution to total variance explained by the model*		
*p*-values and percent contribution	**T**: *p* = 0.001; 11.05% **S**: *p* = 0.001; 20.65% **DO**: *p* = 0.015; 4.11% **Fl**: *p* = 0.015; 5.64% SD: *p* = 0.064; n/a.	**T**: *p* = 0.008; 3.58% **S**: *p* = 0.001; 5.01% DO: *p* = 0.760; n/a **Fl**: *p* = 0.034; 2.61% **SD**: *p* = 0.001; 8.01%

## Data Availability

DNA sequences for this study can be found in the DDBJ Sequence Read Archive under accession number DRA014629 (biosamples SAMD00515557, SAMD00515558, SAMD00515560, SAMD00515561, SAMD00515563, SAMD00515564, SAMD00515566–SAMD00515574, SAMD00515576, SAMD00515577, SAMD00515579–SAMD00515586, SAMD00515588, SAMD00515589, SAMD00515592, SAMD00515593, SAMD00515596, SAMD00515598, SAMD00515599, SAMD00515602, SAMD00515603, SAMD00515606, SAMD00515607, SAMD00515610, SAMD00515611, SAMD00515615, SAMD00515616).

## Supplementary Materials

The following supporting information can be downloaded at: https://www.mdpi.com/article/10.3390/toxins14100685/s1. Table S1: Metadata and parameters of the sampling stations; Table S2: Potentially toxic microalgal species identified in the samples by LM; Table S3: Potentially toxic microalgal species identified in the samples by NGS; Table S4: Sampling stations, filtrated volume for DNA extraction, and qPCR data; Table S5: Calculated LODs for the investigated toxin standards; Table S6: Toxin contents (pg NT^−1^) of the 20–50 µm size fractions of 20 m vertical net tows; Table S7: Toxin contents (pg NT^−1^) of the 50–200 µm size fractions of 20 m vertical net tows; Table S8: Mobile phases and gradients used for LC-FLD measurements; Table S9: Chromatographic settings for LC-FLD; Table S10: Mobile phases and gradients used for various LC-MS/MS measurements; Table S11: Mass spectrometer and chromatographic settings for all LC-MS/MS measurements; Table S12: Investigated lipophilic toxins and domoic acid, including associated quantification and qualification transitions; Table S13: Investigated azaspiracids, including associated quantification and qualification transitions; Table S14: Investigated karlotoxins, including associated quantification and qualification transitions; Table S15: Investigated yessotoxins, including associated quantification transitions. References [37,38,130] are cited in the supplementary materials.
