# Supplementary material for: New Knowledge on Distribution and Abundance of Toxic Microalgal Species and Related Toxins in the Northwestern Black Sea
Source: Toxins (Basel). 2022 Oct 6;14(10):685. doi: 10.3390/toxins14100685 (PMC9610735; doi:10.3390/toxins14100685)
Supplement: Supplementary file 1 [file toxins-14-00685-s001.zip › Figure S2.pdf]

Figure S2: The chemical structures of (A) yessotoxin, (B) 9-Methyl-41a-homoyessotoxin, and (C) 44,55-dihydroxyyessotoxin

A

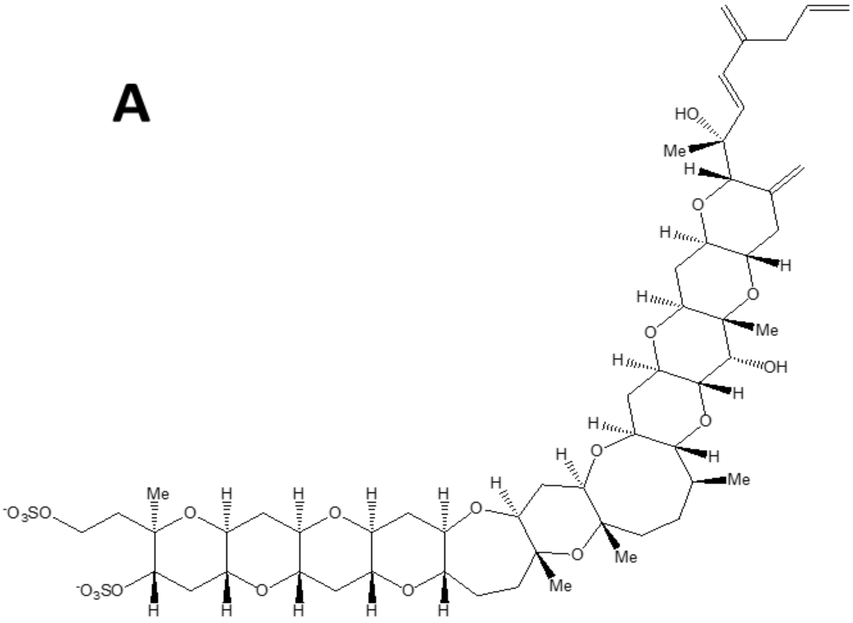

Yessotoxin

B

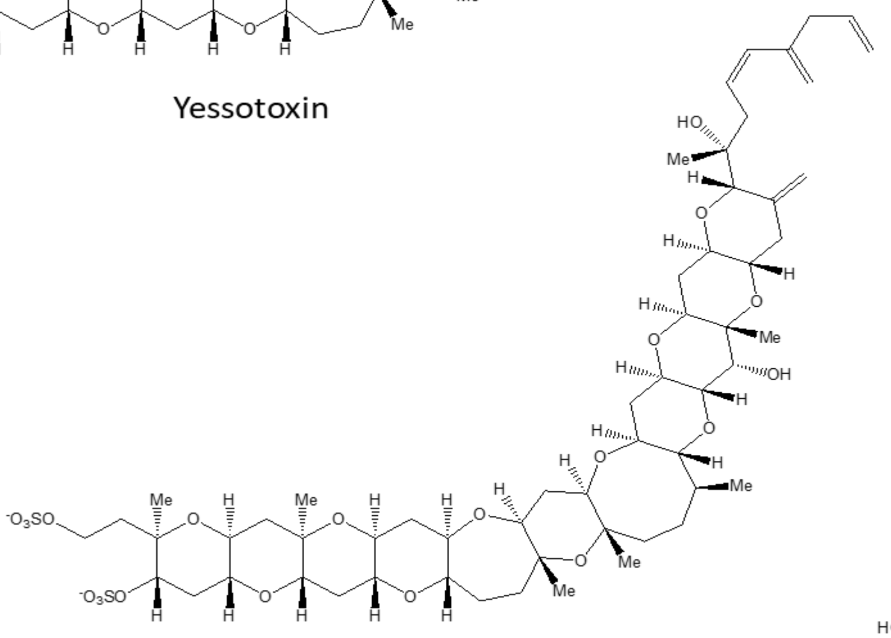

9-Methyl-41a-homoYessotoxin

C

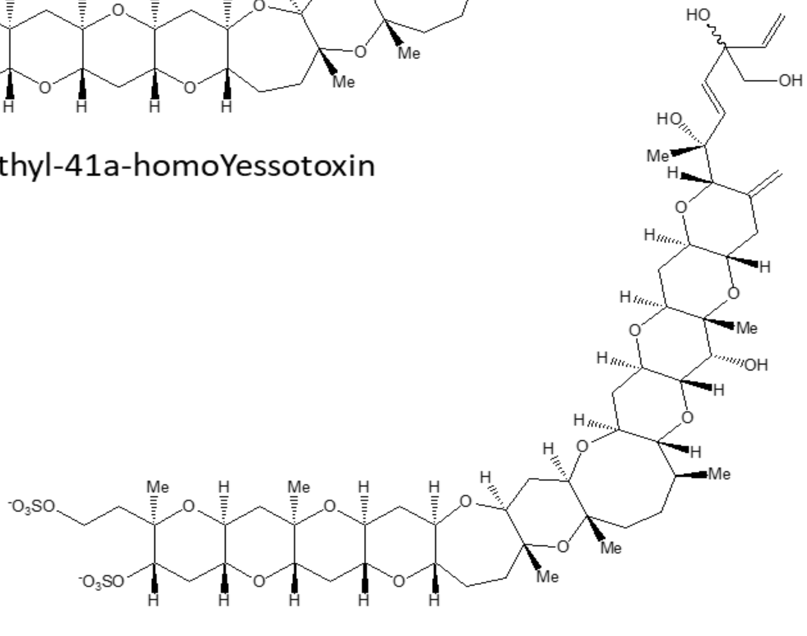

44,55-dihydroxyYessotoxin
