# Supplementary material for: New Knowledge on Distribution and Abundance of Toxic Microalgal Species and Related Toxins in the Northwestern Black Sea
Source: Toxins (Basel). 2022 Oct 6;14(10):685. doi: 10.3390/toxins14100685 (PMC9610735; doi:10.3390/toxins14100685)
Supplement: Supplementary file 1 [file toxins-14-00685-s001.zip › Table S10.pdf]

Table S10. Mobile phases and gradients used for various LC-MS/MS measurements.

	Eluent A	Eluent B	Gradient (linear)		
			Time [min]	%A	Flow [mL min^{-1}]
Lipophilic toxins	aqueous 6.7 mM ammonia	6.7 mM ammonia in ACN/water (9/1 v/v)	0.0	70	0.2
			1.5	10	0.6
			3.5	10	0.6
			4.0	70	0.6
			4.1	70	0.6
			5.0	70	0.6
Domoic acid	50 mM formic acid [1] + 2 mM ammonium formate	50 mM formic acid + 2 mM ammonium formate/acetonitrile (95/5 v/v)	0.0	99	0.2
			5.0	5	0.2
			8.5	5	0.2
			9.0	99	0.2
Azaspiracids	aqueous 6.7 mM ammonia	6.7 mM ammonia in ACN/water (9/1 v/v)	0.0	70	0.2
			1.5	10	0.6
			3.5	10	0.6
			4.0	70	0.6
			4.1	70	0.6
			5.0	70	0.6
Karlotoxins	50 mM formic acid [1] + 2 mM ammonium formate	50 mM formic acid + 2 mM ammonium formate/acetonitrile (95/5 v/v)	0.0	5	0.0
			1.5	5	0.6
			3.5	100	0.6
			6.5	100	0.6
			7.0	5	0.6
Yessotoxins	50 mM formic acid [1] + 2 mM ammonium formate	50 mM formic acid [1] + 2 mM ammonium formate in ACN/MeOH/Water (317/158/25 v/v)	0.0	60	0.3
			6.0	0	0.3
			15.0	0	0.3
			16.0	60	0.3
			22.0	60	0.3

[1] 99%, p.a., Merck
