# Supplementary material for: New Knowledge on Distribution and Abundance of Toxic Microalgal Species and Related Toxins in the Northwestern Black Sea
Source: Toxins (Basel). 2022 Oct 6;14(10):685. doi: 10.3390/toxins14100685 (PMC9610735; doi:10.3390/toxins14100685)
Supplement: Supplementary file 1 [file toxins-14-00685-s001.zip › Table S11.pdf]

Table S11. Mass spectrometer and chromatographic settings for all LC-MS/MS measurements.

**Ion Source**	
Capillary Voltage [kV]	2.00/3.00 [1]
Cone Voltage [kV]	45/40 [1]
Temperature [°C]	600
**Gas flow**	
Evaporation [L h^{-1}]	1000
Cone [L h^{-1}]	150
Nebulizer gas [bar]	7.0
**Others**	
Autosampler temperature [°C]	8
Injection volume [µl]	0.5
Column temperature [°C]	40

[1] Values apply for KmTx Analysis
