# Supplementary material for: New Knowledge on Distribution and Abundance of Toxic Microalgal Species and Related Toxins in the Northwestern Black Sea
Source: Toxins (Basel). 2022 Oct 6;14(10):685. doi: 10.3390/toxins14100685 (PMC9610735; doi:10.3390/toxins14100685)
Supplement: Supplementary file 1 [file toxins-14-00685-s001.zip › Table S12.pdf]

Table S12. Investigated lipophilic toxins and domoic acid including associated quantification and qualification transitions. Toxins marked with a "?" are known but have not yet been fully characterized.

Toxin	Quant.-transition [*m/z*]	Qual.-transition [*m/z*]	Toxin	Quant.-transition [*m/z*]	Qual.-transition [*m/z*]
AZA-1	842 → 824	-	PnTx-E	784 → 164	-
AZA-2	856 → 838	-	PnTx-G	694 → 164	-
AZA-3	828 → 810	-	PnTx-F	766 → 164	-
DA	312 → 266	312 → 193	PTX-2	876 → 213	-
DTX-1	836 → 237	-	PTX-11	892 → 213	-
DTX-2	822 → 223	-	PTX-12	874 → 213	-
GD-A	786 → 733	-	PTX-2sa	894 → 213	-
GYM-A	508 → 490	-	SPX-1	692 → 164	-
GYM-B	524 → 506	-	SPX-A	692 → 150	-
GYM-C	524 → 506	-	SPX-B	694 → 150	-
GYM-D	524 → 506	-	SPX-C	706 → 164	-
GYM-E	526 → 508	-	SPX-D	708 → 164	-
12-Me-GYM-A	522 → 504	-	SPX-G	692 → 164	692 → 150
16-desMe-GYM-D	510 → 492	-	SPX-27-OH-13-desMe-C	694 → 180	-
GYM-?	540 → 522	-	SPX-13,19-didesMe-C	926 → 908	-
GYM-?	542 → 524	-	SPX-20-desMe-D	694 → 150	-
GYM-?	548 → 530	-	20-Me-SPX-G	706 → 164	-
GYM-?	564 → 546	-	YTX	570 → 467	-
GYM-?	582 → 564	-	Homo-YTX	1174 → 979	-
OA	822 → 223	-	45-OH-YTX	1176 → 981	-
OA-d8	946 → 223	-	Homo-45-OH-YTX	1190 → 977	-
