# Supplementary material for: New Knowledge on Distribution and Abundance of Toxic Microalgal Species and Related Toxins in the Northwestern Black Sea
Source: Toxins (Basel). 2022 Oct 6;14(10):685. doi: 10.3390/toxins14100685 (PMC9610735; doi:10.3390/toxins14100685)
Supplement: Supplementary file 1 [file toxins-14-00685-s001.zip › Table S13.pdf]

Table S13. Investigated azaspiracids including associated quantification and qualification transitions. Toxins marked with a "?" are known but have not yet been fully characterized.

**Toxin**	**Quant.-transition [*m/z*]**	**Qual.-transition [*m/z*]**	**Toxin**	**Quant.-transition [*m/z*]**	**Qual.-transition [*m/z*]**
AZA-?	920 → 804	920 → 348	AZA-58	828 → 810	-
AZA-?	928 → 910	928 → 348	AZA-59	860 → 842	-
AZA-1	842 → 824	842 → 672	AZA-60	828 → 810	828 → 348
AZA-2	856 → 838	856 → 672	AZA-62	870 → 852	-
AZA-3 (metabolite)	828 → 810	828 → 658	AZA-1-P	922 → 904	-
AZA-7 (metabolite)	858 → 840	-	AZA-2-P	936 → 918	936 → 802
AZA-11	872 → 854	872 → 362	AZA-7-P	938 → 920	-
AZA-33	716 → 698	-	AZA-11-P	952 → 934	952 → 818
AZA-34	816 → 798	-	AZA-33-P	796 → 778	-
AZA-35	830 → 812	830 → 362	AZA-34-P	896 → 878	-
AZA-36	858 → 840	858 → 348	AZA-35-P	910 → 892	-
AZA-37	846 → 828	846 → 348	AZA-36-P	938 → 920	-
AZA-38	830 → 812	830 → 348	AZA-37-P	926 → 908	-
AZA-39	816 → 798	816 → 348	AZA-38-P	910 → 892	-
AZA-40	842 → 824	-	AZA-39-P	896 → 878	-
AZA-41	854 → 836	854 → 670	AZA-40-P	922 → 904	-
AZA-42	870 → 852	870 → 360	AZA-41-P	934 → 916	-
AZA-43	828 → 810	-	AZA-42-P	950 → 932	-
AZA-50	842 → 824	842 → 348	AZA-50-P	922 → 904	-
AZA-51	858 → 840	858 → 348	AZA-51-P	938 → 920	-
AZA-52	830 → 812	830 → 348	AZA-52-P	910 → 892	-
AZA-53	830 → 812	830 → 348	AZA-53-P	910 → 892	-
AZA-54	870 → 852	-	AZA-54-P	950 → 932	-
AZA-55	868 → 850	868 → 362	AZA-59-P	940 → 922	-
AZA-56	884 → 866	-	AZA-62-P	950 → 932	-
AZA-57	844 → 826	-			
