# Supplementary material for: New Knowledge on Distribution and Abundance of Toxic Microalgal Species and Related Toxins in the Northwestern Black Sea
Source: Toxins (Basel). 2022 Oct 6;14(10):685. doi: 10.3390/toxins14100685 (PMC9610735; doi:10.3390/toxins14100685)
Supplement: Supplementary file 1 [file toxins-14-00685-s001.zip › Table S14.pdf]

Table S14. Investigated karlotoxins including associated quantification and qualification transitions. Toxins marked with a "?" are known but have not yet been fully characterized.

**Toxin**	**Quantitative transition [*m*/*z*]**	**Qualifier transition [*m*/*z*]**
KmTx-?	1238.8 → 839.4	1238.8 → 634.4
KmTx-?	1267.8 → 834.5	-
KmTx-?	1274.8 → 841.5	-
KmTx-10	1303.8 → 877.6	1303.8 → 615.4
KmTx-12	1321.8 → 895.6	1321.8 → 633.4
KmTx-5	1325.8 → 895.6	1325.8 → 691.5
KmTx-?	1342.8 → 1121.7	1342.8 → 719.4
KmTx-3	1347.8 → 937.6	1347.8 → 877.6
KmTx-13	1355.8 → 1319.8	1355.8 → 895.7
KmTx-1	1361.8 → 937.6	1361.8 → 877.6
KmTx-2	1367.8 → 937.6	1367.8 → 877.6
KmTx-11	1379.8 → 877.6	1379.8 → 615.4
AM-18	1381.8 → 1105.6	1381.8 → 687.4
44-oxo-KmTx-2	1383.9 → 937.5	1383.9 → 877.6
Sulfo-KmTx-10	1423.8 → 1303.8	1423.8 → 877.6
