# Supplementary material for: New Knowledge on Distribution and Abundance of Toxic Microalgal Species and Related Toxins in the Northwestern Black Sea
Source: Toxins (Basel). 2022 Oct 6;14(10):685. doi: 10.3390/toxins14100685 (PMC9610735; doi:10.3390/toxins14100685)
Supplement: Supplementary file 1 [file toxins-14-00685-s001.zip › Table S15.pdf]

Table S15. Investigated yessotoxins including associated quantification transitions. All compounds and entries refer to numbering in Miles et al. [37,130].

YTX	570.4 → 467.2
Compounds 17-19	991.5 → 911.5
41-keto-YTX, 40-epi-41-keto-YTX, 41-keto-YTX-enone (compounds 6,7,8)	1047.5 → 967.5
undescribed	1049.5 → 969.5
Entries 21, 22	1061.5 → 981.5
Compound 16	1085.5 → 1005.5
nor-YTX (compound 5)	1101.5 → 1021.5
Entry 17	1117.5 → 1037.5
Undescribed	1131.5 → 1051.5
YTX, entries (32-35)	1141.6→ 1061.6
Entry 37	1143.5 → 1063.5
41a-homo-YTX (compound 5)	1155.5 → 1075.5
45-OH-YTX (compound 2)	1157.5 → 1077.5
Entry 45	1159.5 → 1079.5
9-Me-41a-homo-YTX (compound 10)	1169.5 → 1089.5
undescribed	1171.5 → 1091.5
Carboxy-YTX (compound 3)	1173.5 → 1093.5
44,55-dihydroxy-YTX (compound 13)	1175.5 → 1095.5
Entries 57-60	1187.5 → 1107.5
Compound 14	1189.5 → 1109.5
undescribed	1195.5 → 1115.5
Compound 15	1203.5 → 1123.5
Compound 20	1273.5 → 1193.5
Compound 11	1290.5 → 1210.5
Compound 12	1304.5 → 1224.5
Compound 21	1405.5 → 1325.5

References:

37. Miles, C. O.; Samdal, I. A.; Aasen, J. A. G.; Jensen, D. J.; Quilliam, M. A.; Petersen, D.; Briggs, L. M.; Wilkins, A. L.; Rise, F.; Cooney, J. M.; Lincoln MacKenzie, A. Evidence for numerous analogs of yessotoxin in *Protoceratium reticulatum*. *Harmful Algae* **2005**, *4*, 1075–1091. https://doi.org/10.1016/j.hal.2005.03.005.

130. Miles, C. O.; Wilkins, A. L.; Hawkes, A. D.; Selwood, A. I.; Jensen, D. J.; Munday, R.; Cooney, J. M.; Beuzenberg, V. Polyhydroxylated amide analogs of yessotoxin from *Protoceratium reticulatum*. *Toxicon* **2005**, *45*, 61–71. https://doi.org/10.1016/j.toxicon.2004.09.011.
