# Supplementary material for: New Knowledge on Distribution and Abundance of Toxic Microalgal Species and Related Toxins in the Northwestern Black Sea
Source: Toxins (Basel). 2022 Oct 6;14(10):685. doi: 10.3390/toxins14100685 (PMC9610735; doi:10.3390/toxins14100685)
Supplement: Supplementary file 1 [file toxins-14-00685-s001.zip › Table S4.pdf]

Table S4. Sampling stations, filtrated volume for DNA extraction, and qPCR data (+/+: positive signal in both replicates; nd = not detected.

Station	Date	Latitude [°N]	Longitude [°E]	Sampling depth [m]	Filtered water volume [mL]	qPCR Amphidomataceae	qPCR *Az. spinosum*	qPCR *Az. poporum*	qPCR *Am. languida*
**1**	15.05.2019	43.610	28.700	5, 10, 25	2790	nd	nd	nd	nd
**2**	15.05.2019	43.611	29.374	5, 10, 18	2550	+/+	nd	nd	nd
**3**	16.05.2019	43.601	29.539	5, 15, 25	3450	+/+	nd	nd	nd
**4**	16.05.2019	43.589	30.194	5, 15, 20	2400	+/+	nd	nd	nd
**5**	17.05.2019	44.102	30.229	5, 10, 19	3000	+/+	nd	nd	nd
**6**	17.05.2019	44.122	29.420	5, 10, 25	1350	nd	nd	nd	nd
**7**	18.05.2019	44.119	29.250	5, 12, 25	1300	nd	nd	nd	nd
**8**	18.05.2019	44.304	28.854	0, 12, 16	1680	nd	nd	nd	nd
**9**	19.05.2019	44.313	29.129	5, 10, 15	1640	nd	nd	nd	nd
**10**	19.05.2019	44.299	29.667	0-1	600	nd	nd	nd	nd
**11**	20.05.2019	44.295	29.807	5, 15, 25	1210	nd	nd	nd	nd
**12**	20.05.2019	44.272	30.503	5, 20, 30	1600	+/+	nd	nd	nd
**13**	21.05.2019	44.272	30.658	5, 10, 20	2000	+/+	nd	nd	nd
**14**	21.05.2019	44.396	31.092	5, 15, 31	2000	+/+	nd	nd	nd
**15**	22.05.2019	44.592	31.106	5, 10, 20	1600	+/+	nd	nd	nd
**16**	22.05.2019	44.584	30.450	8, 10, 25	1350	+/+	nd	nd	nd
**17**	23.05.2019	44.600	30.375	5, 12, 25	1000	+/+	nd	nd	nd
**18**	23.05.2019	44.619	29.724	5, 12, 25	1500	+/+	nd	nd	nd
**19**	24.05.2019	44.621	29.551	5, 12, 25	1560	nd	nd	nd	nd
**20**	24.05.2019	44.629	29.142	5, 9, 17	800	nd	nd	nd	nd
**21**	25.05.2019	43.970	28.741	5, 15, 25	2000	nd	nd	nd	nd
**22**	25.05.2019	43.959	29.512	5, 10, 14	1000	nd	nd	nd	nd
**23**	26.05.2019	43.960	29.652	5, 15, 25	1900	nd	nd	nd	nd
**24**	26.05.2019	43.941	30.205	5, 15, 35	1000	nd	nd	nd	nd
**25**	27.05.2019	43.933	30.340	5, 12, 48	1400	nd	nd	nd	nd
**26**	27.05.2019	43.917	31.003	5, 15, 40	1700	nd	nd	nd	nd
**27**	28.05.2019	43.234	30.579	5, 15, 50	2500	nd	nd	nd	nd

**28**	28.05.2019	43.254	29.756	5, 12, 54	2000	nd	nd	nd	nd
**29**	29.05.2019	43.259	29.630	5, 10, 54	2000	nd	nd	nd	nd
**30**	29.05.2019	43.367	29.000	5, 10, 35	2000	nd	nd	nd	nd
**31**	30.05.2019	43.280	28.817	5, 10, 44	2000	nd	nd	nd	nd
**32**	30.05.2019	43.291	28.278	0, 8, 15	1150	nd	nd	nd	nd
**33**	31.05.2019	42.609	27.807	5, 10, 18	1820	nd	nd	nd	nd
**34**	31.05.2019	42.620	28.514	5, 10, 45	2000	nd	nd	nd	nd
**35**	01.06.2019	42.589	28.669	5, 10, 35	1660	nd	nd	nd	nd
**36**	01.06.2019	42.594	29.318	5, 13, 45	2320	nd	nd	nd	nd
**37**	02.06.2019	42.598	29.467	5, 15, 42	3000	nd	nd	nd	nd
**38**	02.06.2019	42.765	29.889	5, 11, 45	2000	nd	nd	nd	nd
**39**	03.06.2019	43.114	28.942	5, 10, 37	2620	nd	nd	nd	nd
**40**	03.06.2019	43.137	28.404	5, 10, 23	2000	nd	nd	nd	nd
**41**	04.06.2019	43.131	28.275	5, 10, 30	2000	nd	nd	nd	nd
