# Supplementary material for: New Knowledge on Distribution and Abundance of Toxic Microalgal Species and Related Toxins in the Northwestern Black Sea
Source: Toxins (Basel). 2022 Oct 6;14(10):685. doi: 10.3390/toxins14100685 (PMC9610735; doi:10.3390/toxins14100685)
Supplement: Supplementary file 1 [file toxins-14-00685-s001.zip › Table S5.pdf]

Table S5. Calculated LODs for the investigated toxin standards.

**PSP toxins by net tow sampling and LC-FLD analysis**	
Toxin	LOD [ng NT^{-1}]
STX	6.08
dc-STX	7.68
NEO	48.39
GTX-1	106.03
GTX-2	5.23
GTX-3	6.23
GTX-4	74.74
B1	26.20
dc-GTX-2	4.64
dc-GTX-3	3.97
**Lipophilic toxins by net tow sampling and LC-MS/MS analysis**	
Toxin	LOD [ng NT^{-1}]
AZA-1	0.12
DA	1.62
DTX-1	37.63
DTX-2	21.42
GD-A	1.86
GYM-A	0.12
OA	84.79
PTX-2	0.39
SPX-1	0.05
YTX	0.03
**AZA and KmTx by water sampling and LC-MS/MS analysis**	
Toxin	LOD [pg L^{-1} sea water]
AZA-1	45.6 – 264.4*
KmTx-2	5,500 – 36,700*

* depending on filtered water volume
