# Supplementary material for: New Knowledge on Distribution and Abundance of Toxic Microalgal Species and Related Toxins in the Northwestern Black Sea
Source: Toxins (Basel). 2022 Oct 6;14(10):685. doi: 10.3390/toxins14100685 (PMC9610735; doi:10.3390/toxins14100685)
Supplement: Supplementary file 1 [file toxins-14-00685-s001.zip › Table S6.pdf]

Table S6. Toxin contents [pg NT^{-1}] of the 20-50 µm size fractions of 20 m vertical net tows. "-" indicates toxin levels below the respective limit of detection (Table S5). PTX-1 and PTX-13 are expressed as PTX-2 equivalents. All YTX variants are expressed as YTX equivalents. [a] YTX numbers refer to Miles et al. [37]. [b] entry numbers refer to Miles et al. [37]. [c] YTX number refers to Miles et al. [38]. [d] vertical net tow from 15 m depth. [e] vertical net tow from 30 m depth. [f] vertical net tow from 40 m depth.

	Pectenotoxins (PTX)			Yessotoxins (YTX)									
**Station**	**PTX-2**	**PTX-1**	**PTX-13**	**YTX**	**#17/18/19 [a]**	**#6/7/8 [a]**	**e# 21/22 [b]**	**#16 [a]**	**#3[c]**	**e# 45 [b]**	**#10 [a]**	**#13 [a]**	**#15 [a]**
1	430	2,140	50	-	-	-	-	-	-	-	-	-	-
2	370	-	90	-	-	-	-	-	-	-	-	-	-
3	24,190	-	1,640	-	-	-	-	-	-	-	-	-	-
4	7,500	-	690	11,850	-	-	-	-	-	-	-	-	-
5	19,250	-	870	-	-	-	-	-	-	-	-	-	-
6	1,930	1,180	-	3,239	-	-	-	-	-	-	-	-	-
7	32,160	1,060	960	6,184	16,093	-	-	-	-	-	-	-	-
8	6,700	-	240	-	-	-	-	-	-	-	-	-	-
9	8,810	1,940	700	-	-	-	-	-	-	-	-	-	-
10	17,970	1,360	1,050	1,753	-	-	-	-	-	-	-	-	-
11	7,890	4,290	300	4,859	-	-	-	-	-	-	-	-	-
12	75,280	7,910	4,910	1,760	-	-	-	-	-	-	-	-	-
13	39,650	-	3,230	-	-	-	-	-	-	-	-	-	-
14	6,900	-	370	-	-	-	-	-	-	-	-	-	-
15	46,110	950	15,290	2,951	-	-	-	-	-	-	-	-	-
16	9,260	2,510	450	-	11,634	5,288	-	-	-	-	-	2,694	6,105
17	2,790	7,530	-	-	-	4,043	-	-	-	-	-	-	-
18	5,930	2,830	-	-	2,672	9,387	-	-	-	-	-	-	-
19	30,960	2,220	960	-	1,900	-	-	-	-	-	-	-	1,381
20	13,550	2,610	230	-	5,050	-	-	-	-	-	-	-	-
21	38,890	13,370	940	12,076	-	-	-	-	-	-	-	-	-
22	95,540	16,590	6,030	2,576	17,962	11,064	-	-	-	4,299	-	-	3,586
23	86,400	18,230	4,960	8,364	1,249	8,759	-	-	-	4,487	-	5,654	4,282
24	18,770	2,690	500	1,251	-	-	-	-	-	-	-	1,618	2,940
25	205,910	2,750	8,440	1,181	-	-	-	-	-	-	-	-	-

26	76,590	1,700	11,290	1,125	-	-	-	-	-	2,321	-	818	6,826
27	12,960	890	710	17,026	-	-	-	-	-	-	-	-	-
28	41,050	400	2,870	13,629	-	-	-	-	-	-	-	-	-
29	25,830	250	1,480	12,522	-	-	-	-	-	-	-	-	-
30	32,640	2,030	1,880	1,472	-	-	-	-	-	-	-	-	-
31	22,150	3,110	740	4,918	-	-	-	-	4,843	662	-	4,715	7,831
32	69,670	11,770	2,150	11,731	-	17,094	2,677	-	4,744	8,398	-	8,782	20,956
33	65,110	33,280	1,980	22,722	-	3,033	2,582	-	2,943	-	-	2,995	7,483
34	47,960	4,600	1,690	9,566	-	-	-	-	-	-	-	-	3,880
35	5,310	590	-	-	-	2,092	-	-	3,395	3,394	-	1,784	5,072
36	21,140	170	730	5,835	-	-	-	-	-	-	-	-	-
37	48,870	440	1,400	32,713	-	-	-	-	-	-	-	-	-
38 [e]	9,050	270	170	10758	-	-	-	-	-	-	-	-	-
39 [e]	28,770	1,400	270	7078	-	-	-	-	-	-	-	-	-
40 [f]	75,100	20,190	1,310	27,798	-	12,930	-	-	6,706	19,949	-	17,415	50,980
41 [f]	48,480	36,500	410	39,706	-	-	5,046	-	-	418	-	-	7,439

References:

37. Miles, C. O.; Samdal, I. A.; Aasen, J. A. G.; Jensen, D. J.; Quilliam, M. A.; Petersen, D.; Briggs, L. M.; Wilkins, A. L.; Rise, F.; Cooney, J. M.; Lincoln MacKenzie, A. Evidence for numerous analogs of yessotoxin in *Protoceratium reticulatum*. *Harmful Algae* **2005**, *4*, 1075–1091. https://doi.org/10.1016/j.hal.2005.03.005.
38. Miles, C. O.; Wilkins, A. L.; Hawkes, A. D.; Selwood, A. I.; Jensen, D. J.; Cooney, J. M.; Beuzenberg, V.; MacKenzie, A. Lincoln. Identification of 45-Hydroxy-46,47-Dinoryessotoxin, 44-Oxo-45,46,47-Trinoryessotoxin, and 9-Methyl-42,43,44,45,46,47,55-Heptanor-38-En-41-Oxoyessotoxin, and partial characterization of some minor yessotoxins, from *Protoceratium reticulatum*. *Toxicon* **2006**, *47*, 229–240. https://doi.org/10.1016/j.toxicon.2005.11.001.
