# Supplementary material for: New Knowledge on Distribution and Abundance of Toxic Microalgal Species and Related Toxins in the Northwestern Black Sea
Source: Toxins (Basel). 2022 Oct 6;14(10):685. doi: 10.3390/toxins14100685 (PMC9610735; doi:10.3390/toxins14100685)
Supplement: Supplementary file 1 [file toxins-14-00685-s001.zip › Table S7.pdf]

Table S7. Toxin contents [pg NT^{-1}] of the 50-200 µm size fractions of 20 m vertical net tows. "-" indicates toxin levels below the respective limit of detection (Table S5). PTX-1 and PTX-13 are expressed as PTX-2 equivalents. All YTX variants are expressed as YTX equivalents. [a] YTX numbers refer to Miles et al. [37]. [b] entry numbers refer to Miles et al. [37]. [c] YTX number refers to Miles et al. [38]. [d] vertical net tow from 15 m depth. [e] vertical net tow from 30 m depth. [f] vertical net tow from 40 m depth.

	Pectenotoxins (PTX)			Yessotoxins (YTX)									
Station	PTX-2	PTX-1	PTX-13	YTX	#17/18/19 [a]	#6/7/8 [a]	e#21/22 [b]	#16 [a]	#3 [c]	e# 45 [b]	#10 [a]	#13 [a]	#15 [a]
1	19,420	38,820	0	-	-	-	-	-	-	-	-	-	-
2	160	370	30	-	-	-	-	-	-	-	-	-	-
3	2,190	0	0	-	-	-	-	-	-	-	-	-	-
4	11,810	0	550	-	-	-	-	-	-	-	-	-	-
5	4,490	0	0	-	-	-	-	-	-	-	-	-	-
6	33,660	34,490	1,010	-	-	-	-	-	-	-	-	-	-
7	64,070	24,750	770	-	-	-	-	-	-	-	-	-	-
8	16,300	14,220	760	-	-	-	-	-	-	-	-	-	-
9	59,970	40,920	3,280	-	-	-	-	-	-	-	-	-	-
10	58,220	13,930	2,210	-	4,330	-	-	-	-	-	-	-	-
11	36,590	40,210	810	-	4,561	-	-	-	-	-	-	-	-
12	8,520	9,040	0	-	-	-	-	-	-	-	-	-	-
13	12,860	240	630	-	-	-	-	-	-	-	-	-	-
14	12,240	770	360	-	-	-	-	-	-	-	-	-	-
15	25,990	6,980	5,150	-	1,155	6,513	-	-	-	-	-	-	-
16	182,570	289,320	0	-	1,530	-	-	-	-	-	-	-	-
17	7,190	127,530	0	-	4,559	-	-	-	-	-	-	-	-
18	333,030	156,870	5,420	-	3,625	-	-	-	-	-	-	-	-
19	70,690	31,110	1,350	-	2,719	-	-	-	-	-	-	-	-
20 [d]	33,680	30,700	900	-	22,281	5,484	-	-	-	-	-	-	-
21	119,670	70,940	2,940	21,556	10,628	3,055	-	-	-	-	-	-	-
22	426,320	335,810	13,990	12,611	36,582	2,780	-	-	-	-	-	-	-
23	94,480	81,860	6,070	-	21,179	66,690	-	-	-	12,747	-	14,764	37,272
24	16,340	10,590	890	-	-	-	-	-	-	-	-	-	-
25	27,750	4,190	2,720	-	-	3,286	-	8,303	-	-	6,353	-	8,694

26	25,680	1,760	3,690	-	-	90,390	-	41,586	-	23,882	22,615	22,861	59,991
27	720	510	0	-	-	-	-	38,402	-	-	14,751	-	-
28	780	0	0	-	-	-	-	28,321	-	-	17,898	-	-
29	530	0	0	-	-	-	-	41,318	-	-	27,230	-	-
30	21,680	1,270	2,120	-	-	-	-	5,012	-	-	4,178	-	-
31	28,610	8,880	1,720	-	-	140,201	-	-	-	39,414	-	28,290	67,903
32	41,100	67,370	2,660	4,405	-	108,811	-	-	-	15,695	-	19,345	26,024
33	51,900	39,610	470	6,087	-	13,126	-	-	-	7,659	-	-	48,940
34	16,030	8,280	370	-	-	-	-	17,670	-	4,033	12,699	-	-
35	7,050	4,510	120	-	-	24,758	-	34,697	-	15,173	19,191	-	31,003
36	10	30	0	-	-	-	-	1,847	-	-	2,044	-	-
37	1,420	130	30	-	-	-	-	4,143	-	-	6,663	-	-
38 [e]	560	60	0	-	-	-	-	26,588	-	-	5,690	-	-
39 [e]	1,630	480	0	-	-	-	-	17,816	-	-	9,038	-	-
40 [f]	14,140	24,100	0	5,603	-	33,662	-	-	-	3,534	-	-	-
41 [f]	77,780	54,700	860	14,054	-	11,248	-	5,162	-	17,757	-	-	40,555

References:

37. Miles, C. O.; Samdal, I. A.; Aasen, J. A. G.; Jensen, D. J.; Quilliam, M. A.; Petersen, D.; Briggs, L. M.; Wilkins, A. L.; Rise, F.; Cooney, J. M.; Lincoln MacKenzie, A. Evidence for numerous analogs of yessotoxin in *Protoceratium reticulatum*. *Harmful Algae* **2005**, *4*, 1075–1091. https://doi.org/10.1016/j.hal.2005.03.005.

38. Miles, C. O.; Wilkins, A. L.; Hawkes, A. D.; Selwood, A. I.; Jensen, D. J.; Cooney, J. M.; Beuzenberg, V.; MacKenzie, A. Lincoln. Identification of 45-Hydroxy-46,47-Dinoryessotoxin, 44-Oxo-45,46,47-Trinoryessotoxin, and 9-Methyl-42,43,44,45,46,47,55-Heptanor-38-En-41-Oxoyessotoxin, and partial characterization of some minor yessotoxins, from *Protoceratium reticulatum*. *Toxicon* **2006**, *47*, 229–240. https://doi.org/10.1016/j.toxicon.2005.11.001.
