# Supplementary material for: New Knowledge on Distribution and Abundance of Toxic Microalgal Species and Related Toxins in the Northwestern Black Sea
Source: Toxins (Basel). 2022 Oct 6;14(10):685. doi: 10.3390/toxins14100685 (PMC9610735; doi:10.3390/toxins14100685)
Supplement: Supplementary file 1 [file toxins-14-00685-s001.zip › Table S8.pdf]

Table S8. Mobile phases and gradients used for LC-FLD measurements.

Eluent A	Eluent B	Gradient (linear)		
		Time [min]	%A	Flow [mL min^{-1}]
40 mM ammonium phosphate[1] + 6 mM 1-octanesulfonic acid[2] + 6 mM 1-heptanesulfonic acid[2], adjusted to pH 7.0 with dilute phosphoric acid[3], + 0.75% tetrahydrofuran[1]	50 mM phosphoric acid + 13 mM 1-octanesulfonic acid, adjusted to pH 7.0 with ammonium hydroxide[1] + 15% (v/v) acetonitrile[1] + 1.5% tetrahydrofuran	0	100	1.0
		15	100	1.0
		16	0	1.0
		35	0	1.0
		36	100	1.0
		45	100	1.0

[1] HPLC grade, Merck; [2] Sigma, Deisenhofen, Germany; [3] p.a., Applichem, Darmstadt, Germany;
