# Supplementary material for: New Knowledge on Distribution and Abundance of Toxic Microalgal Species and Related Toxins in the Northwestern Black Sea
Source: Toxins (Basel). 2022 Oct 6;14(10):685. doi: 10.3390/toxins14100685 (PMC9610735; doi:10.3390/toxins14100685)
Supplement: Supplementary file 1 [file toxins-14-00685-s001.zip › Table S9.pdf]

Table S9. Chromatographic settings for LC-FLD.

General		
Autosampler temperature [°C]	8	
Injection Volume [µl]	20	
Column temperature [°C]	20	
Post-column derivatization		
reagents	Temperature [°C]	Flow [mL min^{-1}]
550 mM ammonium hydroxide + 10 mM periodic acid[2]	50	0.4
0.75 M nitric acid[3]	20	0.4
Detection		
Absorbance wavelength λ_{ex} [nm]	333	
Emission wavelength λ_{em} [nm]	395	

[2] Sigma; [3] p.a., Applichem
