# Supplementary figures and images for: New Knowledge on Distribution and Abundance of Toxic Microalgal Species and Related Toxins in the Northwestern Black Sea
Source: Toxins (Basel). 2022 Oct 6;14(10):685. doi: 10.3390/toxins14100685 (PMC9610735; doi:10.3390/toxins14100685)

**Figure S1:** The chemical structures of (A) pectenotoxin-1, (B) pectenotoxin-2, and (C) pectenotoxin-13

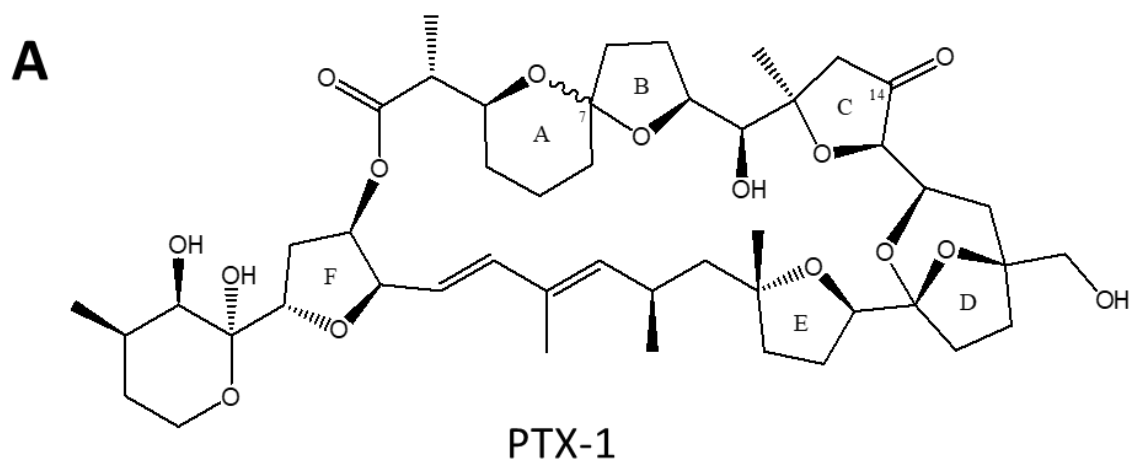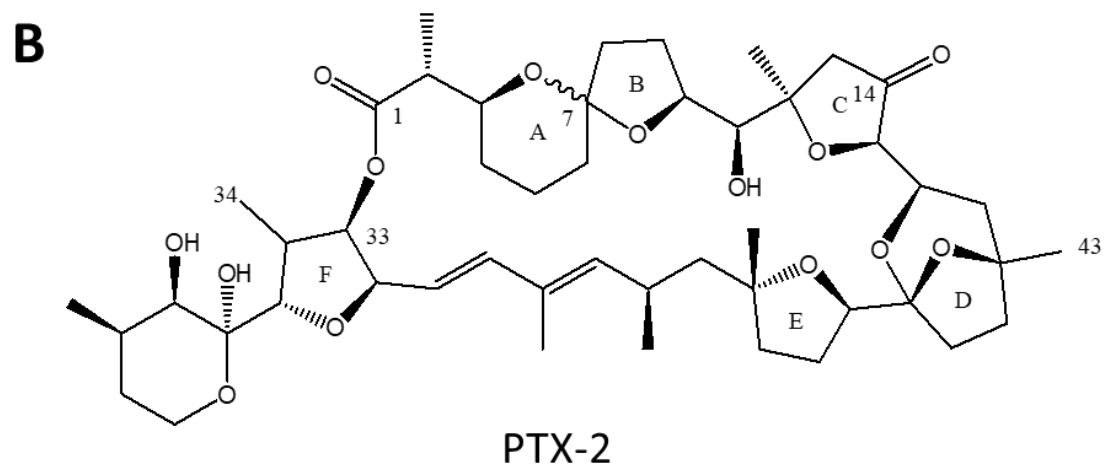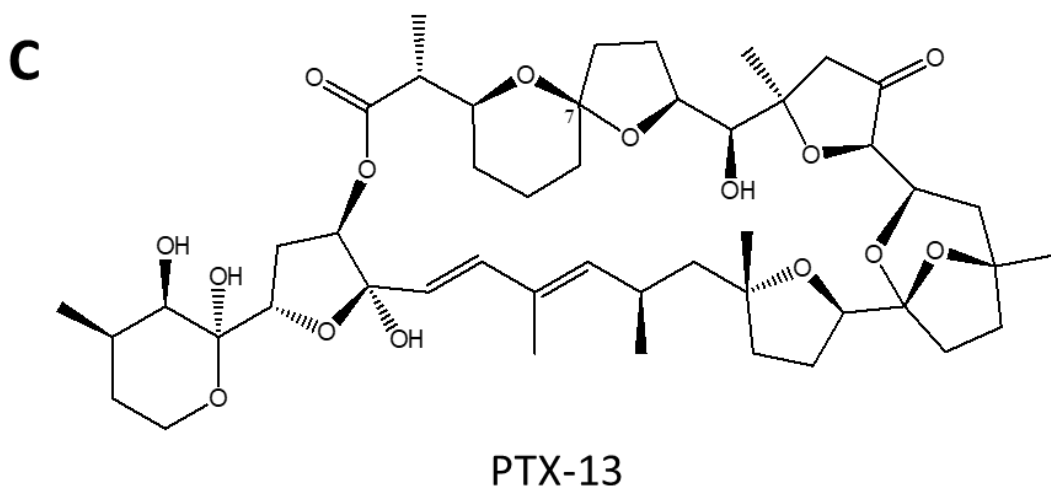

Supplement: Supplementary file 1 [file toxins-14-00685-s001.zip › Figure S1.pdf]
